# Monotropein promotes angiogenesis and inhibits oxidative stress‐induced autophagy in endothelial progenitor cells to accelerate wound healing

**DOI:** 10.1111/jcmm.13434

**Published:** 2017-12-26

**Authors:** Chenggui Wang, Cong Mao, Yiting Lou, Jianxiang Xu, Qingqing Wang, Zengjie Zhang, Qian Tang, Xiaolei Zhang, Huazi Xu, Yongzeng Feng

**Affiliations:** ^1^ Department of Orthopedics The Second Affiliated Hospital and Yuying Children's Hospital of Wenzhou Medical University Wenzhou China; ^2^ Key Laboratory of Orthopedics of Zhejiang Province The Second Affiliated Hospital of Wenzhou Medical University Wenzhou China

**Keywords:** endothelial progenitor cells, angiogenesis, autophagy, wound healing

## Abstract

Attenuating oxidative stress‐induced damage and promoting endothelial progenitor cell (EPC) differentiation are critical for ischaemic injuries. We suggested monotropein (Mtp), a bioactive constituent used in traditional Chinese medicine, can inhibit oxidative stress‐induced mitochondrial dysfunction and stimulate bone marrow‐derived EPC (BM‐EPC) differentiation. Results showed Mtp significantly elevated migration and tube formation of BM‐EPCs and prevented tert‐butyl hydroperoxide (TBHP)‐induced programmed cell death through apoptosis and autophagy by reducing intracellular reactive oxygen species release and restoring mitochondrial membrane potential, which may be mediated *via* mTOR/p70S6K/4EBP1 and AMPK phosphorylation. Moreover, Mtp accelerated wound healing in rats, as indicated by reduced healing times, decreased macrophage infiltration and increased blood vessel formation. In summary, Mtp promoted mobilization and differentiation of BM‐EPCs and protected against apoptosis and autophagy by suppressing the AMPK/mTOR pathway, improving wound healing *in vivo*. This study revealed that Mtp is a potential therapeutic for endothelial injury‐related wounds.

## Introduction

Skin wounds caused by trauma, burns and chronic diseases are a major public health problem around the world, which usually cause pain, infections and even amputation in millions of patients, resulting in decreases in quality of life and heavy medical burden 1, 2. The major goals of wound repair are to shorten the closure time and obtain a functional and aesthetically satisfactory scar. Wound healing is a complex process that is classically divided into three stages, including inflammation, proliferation and remodelling. This process requires the efforts of multiple cells, growth factors and extracellular signals 3. Angiogenesis is critical for wound healing, especially those from chronic and ischemic injuries. Newly formed blood vessels are essential for tissue repair because they can support cells at the wound site with nutrition and oxygen. EPCs, a type of bone marrow mononuclear progenitor cell, can differentiate into endothelial lineage cells and exert vasculogenic effects 4; moreover, endothelial progenitor cells (EPCs) are recruited and stimulated to participate in angiogenesis after tissue wounding 5.

In recent studies, EPC transplantation induced angiogenesis and increased functional blood supply in the ischemic tissues 6. However, clinical studies have reported reduced numbers of EPCs and impaired endothelial function in diabetic patients 7. Furthermore, endothelial injury can be caused by oxidative stress, endoplasmic reticulum stress and damage to the immune system 8, 9. Genetically induced oxidative stress in mice could cause placental angiodysplasia 10. Based on several lines of evidence, oxidative stress induced autophagy upon nutrient deprivation; treatment with antioxidants partially or completely reversed this process 11. Autophagy generally has housekeeping functions, including the removal of dysfunctional components and the recycling of cellular nutrients and energy under stress conditions 12. In contrast, excessive autophagic activities may lead to type II non‐apoptotic programmed cell death due to overconsumption of critical cellular components (also known as autophagic cell death) 13, 14. Furthermore, reactive oxygen species and superoxides generated by mitochondria have been shown to induce autophagy and autophagic cell death 15, 16. Although autophagy has been intensively investigated, the relationships among autophagy, oxidative stress and mitochondrial dysfunction are poorly understood. Hence, researchers have focused not only on enhancing the mobilization and differentiation of EPCs but also on reducing oxidative stress‐induced endothelial dysfunction for therapeutic purposes.

Numerous studies have shown that multiple herbs and herbal extracts of traditional Chinese medicine (TCM) are effective in the treatment of vascular diseases, such as atherosclerosis 17. Monotropein (Mtp) is the primary iridoid glycoside extract from *Morinda officinalis* and has various bioactive effects, including stimulation of osteogenesis in an ovariectomized model 18, anti‐inflammatory activity in osteoarthritis and colitis 19, 20 and anti‐apoptotic and antioxidant effects in hydrogen peroxide‐treated osteoblasts 21. However, the regulation of cellular antioxidants by Mtp is poorly understood. Here, we suggested Mtp may promote angiogenesis and protect against oxidative stress‐induced mitochondrial dysfunction *via* suppression of autophagy.

In this study, the potential angio‐modulatory role of Mtp in bone marrow‐derived EPCs (BM‐EPCs) was studied, and its anti‐apoptotic effects in BM‐EPCs treated with tert‐butyl hydroperoxide (TBHP) to induce oxidative stress and apoptosis were evaluated 22. Furthermore, the possible mechanism underlying the anti‐apoptotic effects of Mtp on BM‐EPCs exposed to oxidative stress and its effects on mitochondrial membrane potential (MMP) in TBHP‐treated BM‐EPCs were investigated. Finally, a full‐thickness cutaneous wound model was used to evaluate the wound healing therapeutic potential of Mtp.

## Materials and methods

### Materials and reagents

Ficoll‐Paque™ PREMIUM was obtained from GE Healthcare (Buckinghamshire, UK). Compound C, 5‐aminoimidazole‐4‐carboxamide‐1‐β‐d‐ribofuranoside (AICAR) and rapamycin were purchased from Selleckchem (Houston, TX, USA). *tert*‐Butyl hydroperoxide (TBHP), 3‐methyladenine (3‐MA), chloroquine (CQ), 2,7‐dichlorodihydrofluorescein diacetate (DCFH‐DA), rhodamine 123, calcium fluorescein‐AM/PI and dimethylsulphoxide (DMSO) were provided by Sigma‐Aldrich (St. Louis, MO, USA). Monoclonal antibodies specific for GAPDH, p‐mTOR, mTOR, p‐4EBP1, 4EBP1 and LC3B were purchased from Cell Signaling Technologies (Beverly, MA, USA). Monoclonal antibodies specific for p‐AMPKα, AMPKα, p‐p70S6K, p70S6K, Bcl‐2, Bax, cleaved‐caspase 3, Beclin‐1, P62, caspase 9, cytochrome c, CD68 and α‐SMA and fluorescein isothiocyanate‐labelled and horseradish peroxidase‐labelled secondary antibodies were purchased from Abcam (Cambridge, UK). Human basic fibroblast growth factor (bFGF) was from Peprotech (London, UK). Crystal violet and 4′,6‐diamidino‐2‐phenylindole (DAPI) were obtained from Beyotime (Shanghai, China). Monotropein (purity ≥98%) was purchased from Herbpurify (Chengdu, China).

### BM‐EPC isolation and identification

All animals were ordered from the SLAC laboratory animal company in Shanghai, China, and all animal procedures were approved by the Wenzhou Medical University Animal Care and Use Committee (approved licence number: wydw2016‐0157). Two‐week‐old male Sprague Dawley (SD) rats were used to isolate the BM‐EPCs in this study. Briefly, rats were killed with an overdose of sodium pentobarbital, and bone marrow‐derived mononuclear cells (BMNCs) were first harvested from the femurs and tibias by Ficoll‐Paque™ PREMIUM density gradient centrifugation of the bone marrow at 2500 r.p.m./min. for 25 min. Mononuclear cells were obtained by carefully collecting solution from the interface between Ficoll‐Paque™ PREMIUM and phosphate‐buffered saline (PBS). After the cells were washed, they were resuspended in EGM‐2MV BulletKit medium and seeded on culture plates pre‐coated with human fibronectin (1 μg/ml) at a density of 3–5 × 10^6^ cells per cm^2^ in a 37°C humidified incubator with 5% CO_2_. One week later, BM‐EPCs were obtained and characterized by DiL‐Ac‐LDL/FITC‐UEA staining and immunofluorescence staining. All BM‐EPCs used in this study were cultured in EGM‐2MV and in passages 3–5.

### Cell culture and treatment protocols

For analysis of the effects of Mtp on apoptosis and senescence in BM‐EPCs, TBHP (100 μM) was used to establish an *in vitro* oxidative stress and apoptosis model. Briefly, cells were pre‐treated with different concentrations of Mtp (0.1, 1, 10, 100 and 1000 μM) for 48 hrs and then with TBHP (100 μM) for 3 hrs. For evaluation of autophagy in BM‐EPCs, the cells were pre‐treated with 100 nM rapamycin (an autophagy agonist), 100 μM 3‐MA (an autophagy inhibitor), 50 μM CQ (another autophagy inhibitor), 5 μm compound C (an AMP‐activated protein kinase inhibitor) or 1 mM AICAR (an AMP‐activated protein kinase agonist) for 2 hrs, respectively, prior to addition of Mtp and TBHP. Mtp was dissolved in DMSO and stored at a concentration of 500 mM at −20°C, and bFGF was used as the positive control. All experiments were performed in triplicate.

### Assessment of cellular proliferation and migration (chemotaxis)

Cell viability was evaluated by Cell Counting Kit‐8 (CCK‐8; Dojindo Co., Japan) assays according to the instructions. In brief, BM‐EPCs were seeded onto 96‐well plates (5000 cells/cm^2^) at 37°C for 24 hrs. For the proliferation assay, cells were treated with different concentrations of Mtp (0.1, 1, 10, 100 and 1000 μM) for 48 hrs. For the cell viability assay, cells were treated with Mtp (100 μM), 3‐MA, CQ, compound C and AICAR, respectively, as described above, followed by incubation with 100 μM TBHP for 3 hrs to induce oxidative stress. After treatment, 10 μl of tetrazolium substrate was added, and the plates were cultured for 1 hr. The absorbance was measured at 450 nm using a microplate reader (Thermo Scientific, Multiskan Go).

Live/dead staining was also used to determine the cell viability by performing a double staining assay using calcium fluorescein‐AM/PI. After 48 hrs pre‐treatment of Mtp, cells were treated with or without 100 μM TBHP for 3 hrs, and BM‐EPCs were then gently washed twice with PBS, 2 μM of calcein‐AM and 15 μg/M PI were added to the cells, and culture plates were incubated at 37°C for 30 min. Finally, the dye solution was removed, and the samples were washed with PBS three times. A fluorescence microscope (Nikon) was used to assess the slides.

A scratch assay and a transwell assay were performed to investigate the migratory activity of BM‐EPCs following Mtp treatment. For the scratch assay, 5 × 105 BM‐EPCs were cultured on 6‐well plates pre‐coated with human fibronectin for 12 hrs. A 200‐μl pipette tip was used to prepare a cell‐free gap on the confluent cells. After washing, cells were treated with different concentrations of Mtp (1, 10 and 100 μM) and bFGF (50 ng/ml) for 12, 48 or 120 hrs. Wound closure was assessed by measuring the size of the cell‐free gap in the wound area for five replicates per group. For transwell assay (Corning, 8 μm, USA), 1 × 10^5^ cells were seeded on the upper chamber, and the lower chamber contained culture medium with 1% FBS and different concentrations of Mtp. BM‐EPCs migrated for 12 hrs in a 37°C cell culture incubator. For quantification of the BM‐EPCs migrating on the membrane, the membrane of the upper chamber was carefully removed, washed and fixed with 4% paraformaldehyde for 30 min. at room temperature. The membrane was then removed and stained with crystal violet. The migrated cells were examined using a fluorescence microscope (Nikon, ECLIPSE Ti, Japan). All experiments were performed three times, and 10 random fields of EPCs were counted for statistical analysis.

### Cell adhesion assay

For cell–matrix adhesion assay, the cell–matrix adhesion assay was performed as previously described 23. Briefly, BM‐EPCs (1 × 10^4^ cells/well) were seeded into culture plates pre‐coated with fibronectin and incubated at 37°C for 30 min. followed by washing with PBS three times. Adherent cells were then fixed and stained with Hoechst 33258 (Beyotime, Shanghai, China). Five random fields were visualized and counted under a fluorescence microscope (Nikon).

To analyse the effects of Mtp on the functional properties of intercellular adhesion, a cell–cell adhesion assay was performed as described 23. Briefly, confluent BM‐EPCs were first stained with Hoechst 33258. Another group of BM‐EPCs was pre‐treated with Mtp (100 μM) for 48 hrs, followed by treatment with calcein‐AM, a cytomembrane tracker, for 30 min. at 37°C, and then seeded onto the Hoechst 33258‐labelled cell monolayer (acceptor cells). After 20 min., the attachment and spread of seeded cells that remained attached after three gentle washes with PBS were monitored and recorded with a fluorescence microscope (Nikon).

### *In vitro* tube formation assay

A tube formation assay on Matrigel (BD Biosciences, USA) was performed to evaluate the effects of Mtp on BM‐EPC morphogenesis and tube formation capacity. Briefly, Matrigel solution was thawed at 4°C overnight and then placed in a 96‐well plate (50 μl per well) in a cell incubator for 1 hr to solidify. A total of 1 × 10^5^ cells per well were then seeded in the Matrigel‐pre‐coated 96‐well plate. Tube formation was observed and quantified, and five independent fields were counted for an average number under an inverted light microscope (Nikon).

### Reactive oxygen species (ROS) production

#### Intracellular ROS evaluation

ROS levels of BM‐EPCs induced by TBHP were measured using the fluorescent dye DCFH‐DA. Cells were incubated with DCFH‐DA (10 μM) at 37°C for 30 min. and then washed with PBS three times. Images of DCFH‐DA fluorescence were immediately recorded using an inverted fluorescence microscope (Nikon).

#### Superoxide measurement using dihydroethidium (DHE)

In the presence of ROS or superoxide anions, DHE is oxidized to ethidium bromide (EtBr), which is characterized by red fluorescence, and intercalates into the DNA. Thus, the amount of EtBr detected by fluorescence microscopy correlates with cellular ROS levels. Briefly, cells were incubated with DHE (10 μM) for 30 min. at 37°C. Images of DHE fluorescence were immediately recorded and quantified under an inverted fluorescence microscope (Nikon).

### Assessment of MMP

MMP (ΔΨm) was measured using rhodamine 123, a cationic lipophilic fluorochrome that can be absorbed by mitochondria. After incubation for 30 min. in the dark, the cells were washed twice, and fluorescence images were immediately recorded and quantified under an inverted fluorescence microscope (Nikon).

### Terminal deoxynucleotidyl transferase (TdT) dUTP nick end labelling (TUNEL)

The TUNEL method is a useful technique for measuring apoptotic DNA fragmentation. BM‐EPCs were seeded on cell culture slides in a six‐well plate and allowed to attach for 24 hrs, followed by Mtp treatment for 48 hrs and TBHP for 2 hrs. EPCs were fixed, permeabilized with 0.1% Triton X‐100 for 5 min. and washed. Cells were then stained using the DeadEnd™ Fluorometric TUNEL System (Promega, USA) and DAPI according to the manufacturers’ instructions. Apoptotic changes were measured using a fluorescence microscope (Leica, DM 2500, Germany).

### RNA isolation, cDNA synthesis and quantitative real‐time PCR (qRT‐PCR)

Total RNA extracts were obtained using TRIzol reagent (Invitrogen), and 1 μg of total RNA was used to synthesize cDNA (TaKaRa, Japan). qPCR was performed using the SYBR Green system (Bio‐Rad, Hercules, USA). Amplification of cDNA samples was carried out at 95°C for 3 min. followed by 40 cycles of 95°C for 15 sec. and 60°C for 45 sec. on an Applied CFX96^®^ real‐time PCR system (Bio‐Rad). The relative quantification of target genes was normalized to housekeeping gene β‐actin, and target genes were compared to each corresponding target gene from the control sample using the 2^−ΔΔCT^ method. The primers for VEGF‐A, KDR, PECAM‐1 and β‐actin are listed as follows: VEGF‐A (F) 5′‐AGCGGAGAAAGCATTTGTTTG‐3′, (R) 5′‐AACGCGAGTCTGTGTTTTTGC‐3′; KDR (F) 5′‐CACCATGCAGACGCTGACAT‐3′, (R) 5′‐TCTAGCTGCCAGTACCATTGGA‐3′; PECAM‐1 (F) 5′‐GCCCTGTCAC GTTTCAGTTT‐3′, (R) 5′‐CCACGGAGCAAGAAAGACTC‐3′; VE‐cadherin (F) 5′‐GTAACCCTGTAGGGAAAGAGTCCATT‐3′, (R) 5′‐GCATGCTCCCGATTAAA CTGCCCATA‐3′; β‐actin (F) 5′‐AACACCCCAGCCATGTACGTA‐3′, (R) 5′‐TCT CCGGAGTCCATCACAATG‐3′.

### Western blot analysis

Samples containing 30 μg of protein were separated on SDS‐PAGE. After being transferred to PVDF membranes, proteins were incubated with primary antibodies overnight, followed by incubation with horseradish peroxidase‐conjugated secondary antibodies for 2 hrs. Bands were detected by electrochemiluminescence reagent, and band intensity was quantified using Image Lab 3.0 software (Bio‐Rad).

### Establishment of a wound model

Twenty SD rats were anaesthetized with 2.5% pentobarbital sodium (30 mg/kg). After the rats were shaved and sterilized, two full‐thickness wounds (20 mm in diameter) were made on each side of the rat's back and were then covered with petrolatum gauze and a sterile bandage. After surgery, the animals were immediately injected with Mtp (25 mg/kg i.p.), and given daily 25 mg/kg doses until they were killed. The control group was injected with an equivalent dose of normal saline. Following treatment with Mtp or normal saline, animals were treated uniformly until the final analysis of the data. All animals showed no significant side effects resulting from drug treatment such as mortality or signs of infectious disease during the experiments. These rats were housed in individual cages, and images of the wounds were taken every 7 days. Wound area was calculated by tracing the wound margins and was evaluated as a per cent area of the original wound using Image‐Pro Plus 6.0 software.

### DiL‐Ac‐LDL‐labelled EPC recruitment in the wound site

To evaluate the recruitment of EPCs at the wound site, the cells were labelled with DiL‐Ac‐LDL (Sigma‐Aldrich, St. Louis, MO, USA). After wounding, DiL‐Ac‐LDL‐positive EPCs were injected intravenously and the wounds were harvested on day 7 and embedded for cutting of frozen sections, which were stained with DAPI as described above. A total of 20 different granulation tissue fields (four sections from each animal) were selected, and the DiL‐labelled EPCs were counted.

### Histological analysis

Tissue samples were collected on days 7, 14 and 21 for histological analysis. After fixation in 4% paraformaldehyde for 48 hrs, samples were embedded in paraffin, sectioned (5 μm) and then stained with haematoxylin and eosin (H&E) and for collagen formation by Masson's trichrome staining. Slides were observed under a microscope. Numbers of macrophages and capillaries were determined to evaluate wound healing conditions.

### Immunofluorescence

For cells, BM‐EPCs were seeded on cell culture slides in a six‐well plate and allowed to attach for 24 hrs and were then treated with different concentrations of Mtp for 48 hrs and TBHP for 3 hrs. For Bcl‐2, Bax and LC3B staining, the samples were fixed with 4% paraformaldehyde and permeabilized in PBS containing Triton X‐100 for 10 min. After the samples were blocked with 10% bovine serum albumin at 37°C for 1 hr, the slides were incubated with primary antibodies against Bcl‐2 (1:200), Bax (1:200) or LC3 (1:200) at 4°C overnight. Then, the slides were washed and incubated with fluorescein isothiocyanate‐ or tetramethyl rhodamine isothiocyanate‐conjugated secondary antibodies for 1 hr at 37°C and labelled with DAPI for 1 min. at room temperature. Finally, three fields of view were randomly selected for observation on each slide with a fluorescence microscope (Olympus Inc., Tokyo, Japan), and the staining intensity was measured using Image‐Pro Plus 6.0 by observers who were blinded to the experimental groups.

For tissues, 5‐μm‐thick skin tissue sections were deparaffinized, rehydrated and treated with the following primary antibodies: anti‐CD68 (1:200) and anti‐α‐SMA (1:200), before being washed four times with PBS and incubated with Alexa Fluor 568 or Alexa Fluor 488 donkey anti‐rabbit/mouse secondary antibodies for 1 hr at 37°C. Then, the sections were washed with PBS, incubated with DAPI for 1 min., rinsed with PBS and finally sealed with a coverslip. All images were captured on a confocal fluorescence microscope (Nikon, Japan).

### Assessment of blood flow in the wound area

The blood flow in wound area was assessed by a laser Doppler imager (MoorLDI‐2, Moor Instruments Limited, Devon, UK) with a laser wavelength of 633 nm, scan of 55 cm and scan duration of 5 min. at 25 ± 5°C. Briefly, the rats were anaesthetized, shaved and then gently fixed onto a black platform. MoorLDI Review V6.1 software was used to quantify the results.

### Statistical analysis

Numerical data from at least three individual experiments are shown as the mean ± SD unless otherwise indicated. The data were analysed by one‐way analysis of variance (ANOVA, Tukey's post hoc analysis) using GraphPad Prism 7.0 (La Jolla, CA, USA). Statistical significance was set at *P < 0.05, **P < 0.01, ***P < 0.005 and ****P < 0.001 *versus* the indicated group.

## Results

### BM‐EPC phenotypes

Rat BM‐EPCs were successfully isolated from the femurs and tibias of 2‐week‐old SD rats. In the initial week, proliferative endothelial colonies, indicative of BM‐EPCs, were observed (Fig. S1a). In contrast, BM‐EPCs cultured for over 2 weeks showed a cobblestone‐like morphology and lacked cluster‐like formations. After coculture with DiL‐Ac‐LDL, positive staining for DiL‐Ac‐LDL and UEA lectin was observed in BM‐EPCs (Online Resource 1: Fig. S1b). Strong expression of BM‐EPC markers (CD31 and KDR) confirmed their endothelial cell characteristics (Fig. S1c). The tube formation assay results revealed their angiogenic ability *in vitro* (Fig. S1d). These results indicated that cells derived from bone marrow (passages 3 to 5) were predominantly BM‐EPCs and maintained both endothelial characteristics and differentiation potential.

### Mtp promotes proliferation, migration, recruitment and tube formation ability of BM‐EPCs

**Figure 1:**
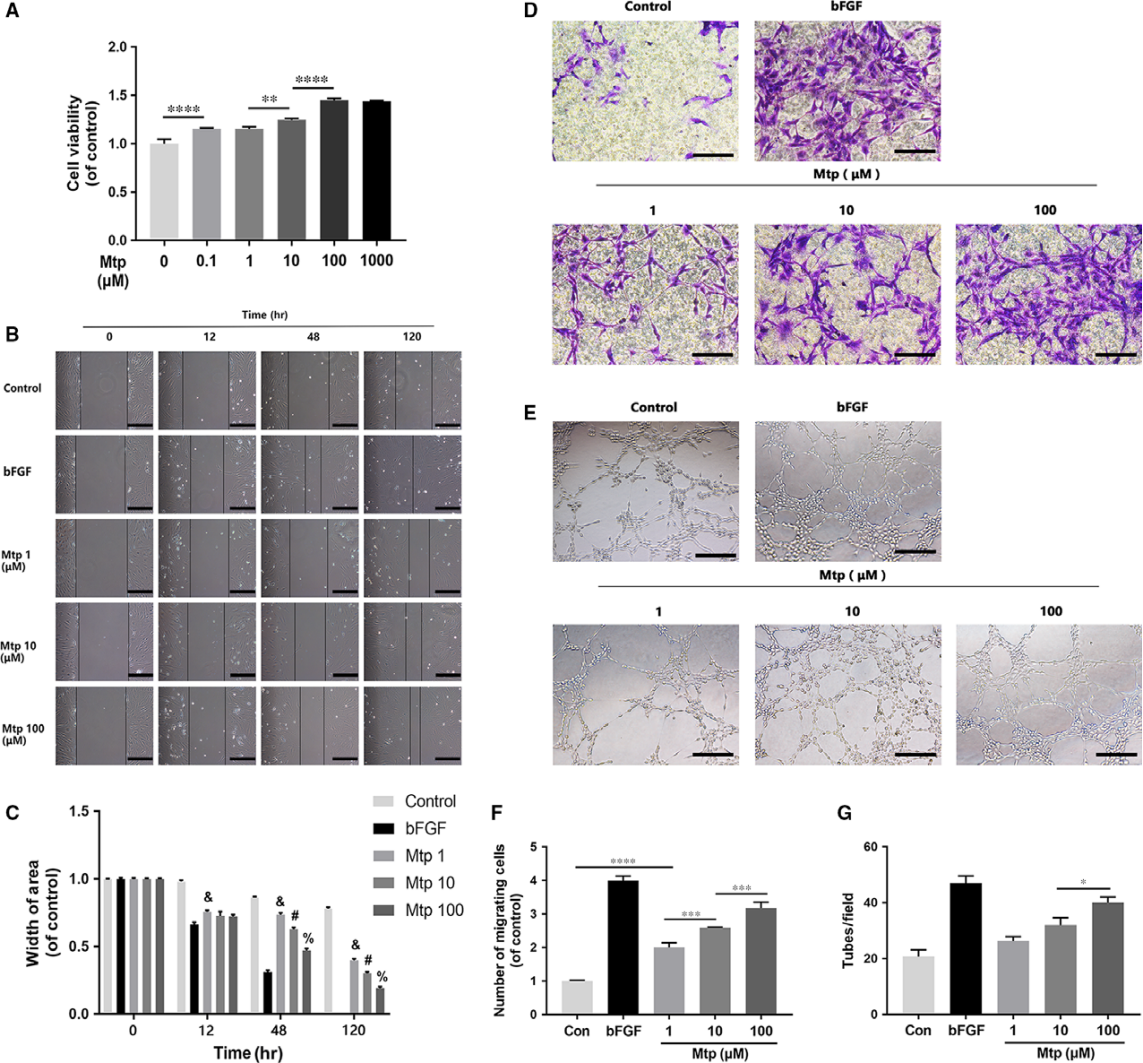
Effect of Mtp on cellular proliferation, migration, recruitment and tube formation of BM‐EPCs. (**A**) Cell proliferation results of BM‐EPCs treated with different concentrations of Mtp for 48 hrs. Cells proliferated evidently faster after Mtp treatment; (**B**,** C**) cell migration regulated by Mtp treatments. Scratch assay showed that EPCs migrated evidently faster in the Mtp‐treated group (scale bar: 200 μm); Data are presented as mean ± SD, & *P* < 0.05 versus the control group, # *P* < 0.05 versus the 1 μM Mtp treated group, % *P* < 0.05 versus the 10 μM Mtp treated group; (**D**,** F**) transwell chemotaxis assay results of BM‐EPCs with different treatments. BM‐EPCs were treated with PBS (control), 50 ng/ml bFGF and 1, 10 and 100 μM Mtp in the lower chamber for 3‐hrs incubation. Numbers of migrated cells were quantified by counting cells in 10 random fields using an inverted microscope (scale bar: 50 μm). The migration of BM‐EPCs was enhanced after Mtp treatment; (**E**,** G**) *in vitro* tube formation results of BM‐EPCs treated by Mtp. Cells were grown on Matrigel™ for 6 hrs under normal growth conditions, five independent fields were assessed for each well and the number of tubes were determined (scale bar: 100 μm). The tube formation ability of BM‐EPCs was improved after Mtp treatment. *n* = 3 independent experiments. *P < 0.05, **P < 0.01, ***P < 0.005, and ****P < 0.001 versus the indicated group.

CCK‐8 test was used to assess whether Mtp affected the proliferation of BM‐EPCs. Results showed significant increases in cell viability at 0.1, 1, 10, 100 and 1000 μM compared with the untreated group (Fig. 1A), which indicated cell proliferation was promoted by the treatment of Mtp. However, no significant effects on cellular proliferation were observed between Mtp concentrations of 0.1 and 1 μM as well as 100 and 1000 μM. A scratch assay was used to investigate the cell migration influenced by Mtp, and bFGF was used as the positive control. When cocultured with Mtp, BM‐EPCs showed enhanced migration into the scratched region (Fig. 1B and C) with an accelerated migration rate at 24 hrs, which became more pronounced at 48 and 72 hrs. Notably, 100 μM Mtp group exhibited a faster migration speed than the groups treated with other concentrations at 48 and 72 hrs.

A chemotaxis assay was performed to test cell recruitment affected by Mtp. The migration ability of BM‐EPCs increased in a dose‐dependent manner (Fig. 1D and F) when treated with different concentrations of Mtp. Much more numbers of migrating BM‐EPCs were found compared with control, which became more significant in 10 and 100 μM Mtp pre‐treatment groups. Furthermore, tube formation ability was evaluated through an *in vitro* angiogenesis assay. As shown in Fig. 1E and G, after incubation on Matrigel for 3 hrs, tubule numbers in the 1, 10 and 100 μM Mtp‐treated groups were significantly increased compared with controls. Additionally, Mtp groups had more mature and better morphology tubes. Therefore, Mtp potently enhanced *in vitro* tube formation ability of BM‐EPCs.

### Mtp regulates adhesion of BM‐EPCs

**Figure 2:**
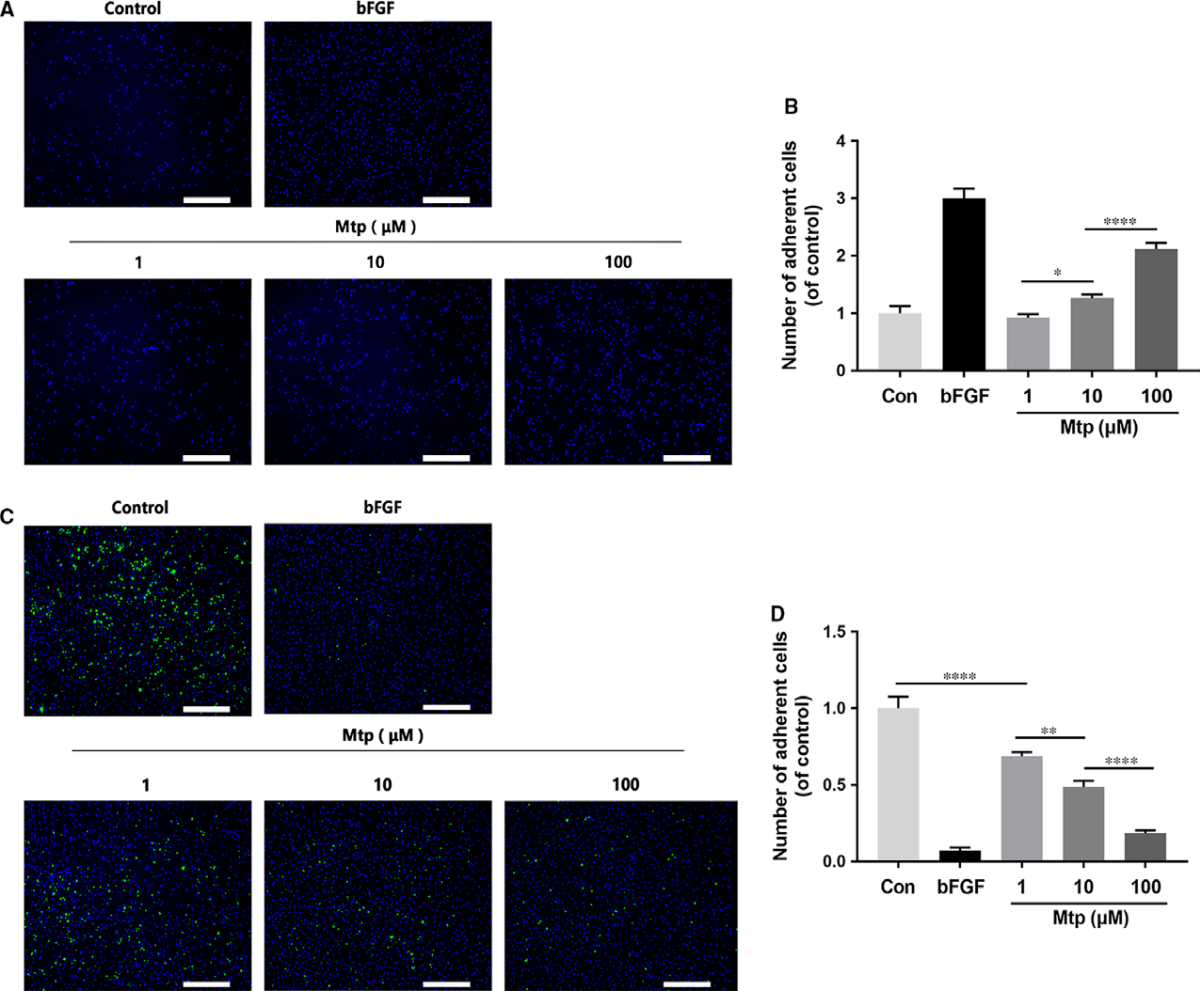
Mtp increases cell–matrix adhesion and decreases cell–cell adhesion. (**A**–**D**) Cell–matrix adhesion assay results of BM‐EPCs treated with Mtp. Cell–cell adhesion assay using Hoechst 33258 dye and calcein‐AM staining exhibited that Mtp treated for 48 hrs markedly increased cell–matrix adhesion and decreased cell–cell adhesion (scale bar: 500 μm). *n* = 3 independent experiments. *P < 0.05, **P < 0.01, ***P < 0.005, and ****P < 0.001 versus the indicated group.

Vasculogenesis and angiogenesis are complex multistep processes that involve remodelling of the ECM and migration and proliferation of endothelial cells. Here, human fibronectin was used as an ECM substitute to study the effect of Mtp on cell–matrix adhesion. Results showed Mtp‐treated cells attached to the ECM more easily than control cells (Fig. 2A and B). For cell–cell adhesion, Mtp treatment significantly reduced adherence to the BM‐EPCs monolayer at 1 μM, and this phenomenon peaked at 100 μM (Fig. 2C and D). Thus, Mtp augmented cell adhesion to the ECM but reduced cell–cell adhesion capacity.

### Mtp up‐regulates the mRNA levels of angiogenic markers and induces angiogenic differentiation *via* mTOR/p70S6K/4EBP1 signalling

**Figure 3:**
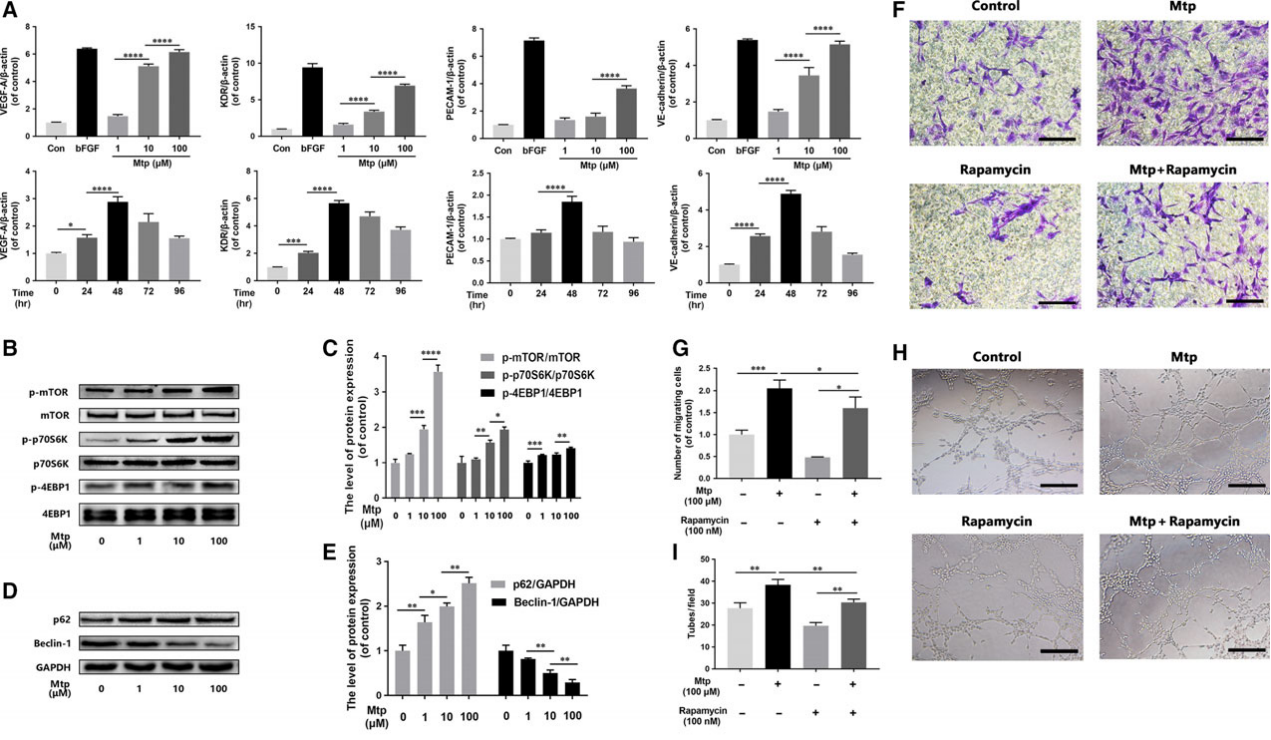
mTOR pathway regulates chemotaxis and capillary tube formation capacity of BM‐EPCs. (**A**) Gene expression of VEGF, KDR, PECAM‐1 and VE‐cadherin in Mtp‐treated BM‐EPCs. Cells were cultivated with 1, 10 and 100 μM Mtp or bFGF (50 ng/ml) for 48 hrs and treated with 100 μM Mtp for 24, 48, 72 and 96 hrs. Gene levels were assessed *via* qRT‐PCR and normalized to β‐actin; related gene was up‐regulated by Mtp at 48 hrs or less; (**B**–**E**) Western blot analysis of p‐mTOR, p‐p70S6K, p‐4EBP1, SQSTM1/P62 and Beclin‐1 in different doses of Mtp‐treated BM‐EPCs for 48 hrs. Mtp evidently increased mTOR pathway proteins and decreased autophagy level; (**F**,** G**) cell chemotaxis regulated by the treatments of rapamycin and/or Mtp. BM‐EPCs were treated with 100 nM rapamycin for 2 hrs prior to treatment with Mtp for 48 hrs. The numbers of migrated cells were quantified by performing cell counts of 10 random fields (scale bar: 50 μm); (**H**,** I**) *in vitro* tube formation results of BM‐EPCs treated by rapamycin and/or Mtp (scale bar: 100 μm); the densitometric analysis of all Western blot bands was normalized to the total proteins or GAPDH. *n* = 3 independent experiments. *P < 0.05, **P < 0.01, ***P < 0.005, and ****P < 0.001 versus the indicated group.

As shown in Fig. 3A, Mtp treatments led to increased levels of VEGF, KDR, PECAM‐1 and VE‐cadherin mRNA in a concentration‐dependent manner with peak increase at 100 μM Mtp‐treated group compared with controls. Interestingly, the increased mRNA levels of VEGF, KDR, PECAM‐1 and VE‐cadherin were not time‐dependent; maximum enhancement was observed at 48 hrs. Based on these data, 100 μM Mtp up‐regulated genes involved in angiogenesis and promote the differentiation of EPCs, and the strongest effects on gene activation were observed 48 hrs after treatment.

To investigate the molecular mechanisms of cellular processes, particularly angiogenesis, Western blot was used to analyse the related proteins. Mtp effectively increased the phosphorylation of mTOR and its two target kinases, p70S6K and 4EBP1, in BM‐EPCs in a dose‐dependent manner (Fig. 3B and C). Moreover, the autophagy flux markers SQSTM1/P62 were up‐regulated and Beclin‐1 was substantially down‐regulated by Mtp treatments (Fig. 3D and E), showing the decreased autophagy level by the Mtp treatment. Furthermore, cell migration and tube formation were notably suppressed after BM‐EPCs were cotreated with rapamycin (Fig. 3F–I), a specific mTOR agonist, suggesting that Mtp enhanced mobility and angiogenesis of BM‐EPCs by activating the mTOR signalling pathway.

### Mtp attenuates intracellular ROS accumulation‐induced apoptosis

**Figure 4:**
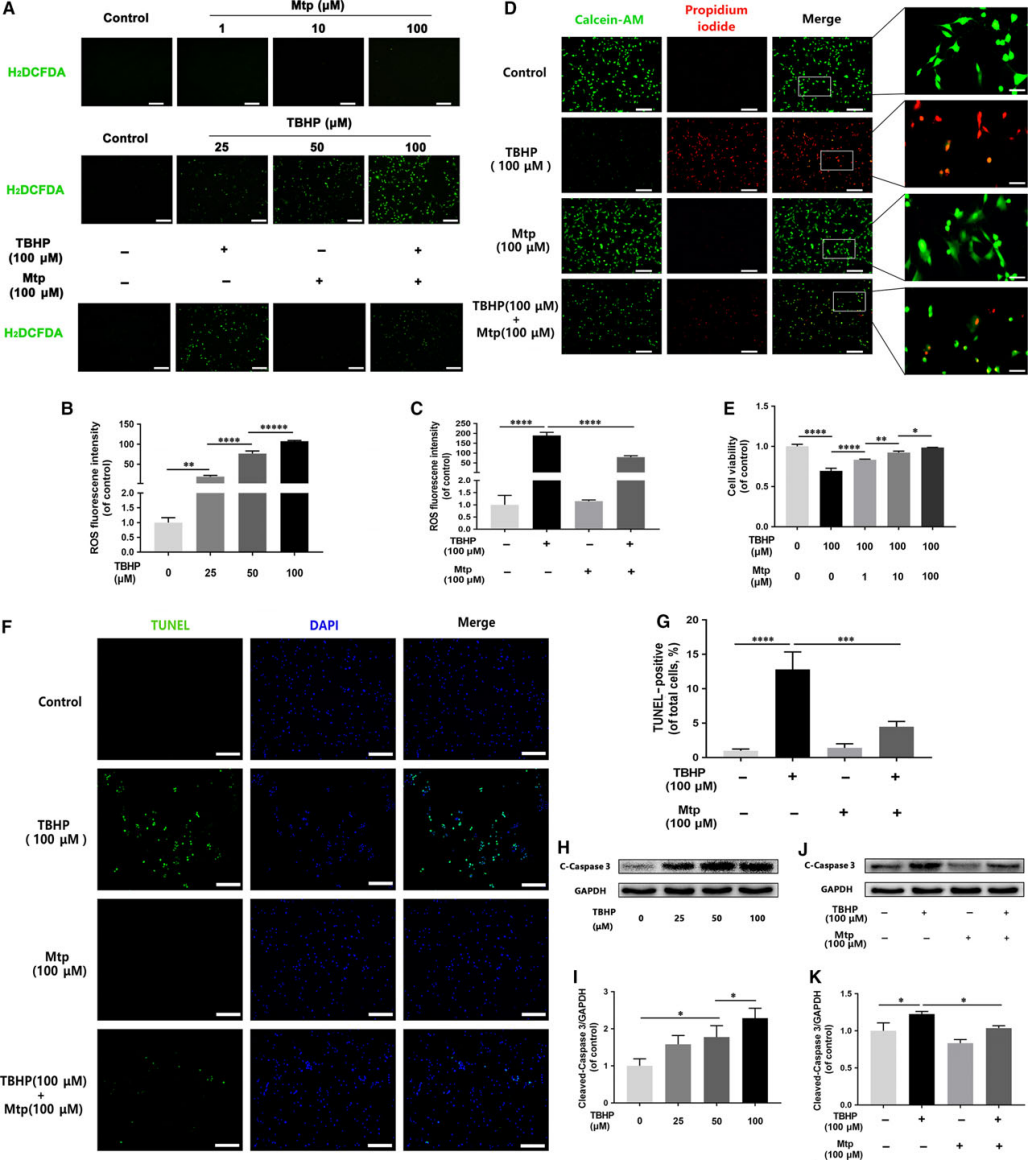
Mtp declines TBHP‐induced apoptosis and ROS production in BM‐EPCs. (**A**–**C**) ROS production detected by H_2_ DCFDA fluorescence in BM‐EPCs. Cells were treated with 1, 10 and 100 μM Mtp for 48 hrs, labelled with H_2_ DCFDA (30 μM) and then incubated with 25, 50 and 100 μM TBHP for 3 hrs prior to fluorescence microscopic analysis (scale bar: 200 μm). Mtp significantly attenuated TBHP‐induced ROS production; (**D**) live/dead staining results of cells treated by Mtp and/or TBHP. Cells were treated with 1, 10 and 100 μM Mtp for 48 hrs and TBHP for 3 hrs, followed by calcein‐AM/PI double staining. Cell survival was significantly up‐regulated by the preconditioning of Mtp even with TBHP treatment (scale bar: 200; 20 μm); (**E**) cell viability results of BM‐EPCs treated with Mtp and TBHP. Cell Counting Kit‐8 (CCK‐8) assay of BM‐EPCs pre‐treated with 1, 10 and 100 μM Mtp for 48 hrs followed by TBHP stimulation was performed, and cell viability was evidently increased by the Mtp pre‐treatment; (**F**,** G**) TUNEL assay was performed in BM‐EPCs as pre‐treated with 100 μM Mtp followed by TBHP stimulation, and Mtp ameliorates apoptosis of BM‐EPCs induced by TBHP (scale bar: 200 μm); (**H**,** I**) Western blot analysis results of levels of cleaved‐caspase 3 in BM‐EPCs with different doses of TBHP treatment. BM‐EPCs were treated with 25, 50 and 100 μM TBHP. The protein expression of cleaved‐caspase 3 was significantly increased after TBHP treatment; (**J**,** K**) Western blot analysis results of expression of cleaved‐caspase 3 after Mtp pre‐treatment. Cells pre‐treated with 100 μM Mtp followed by TBHP stimulation. Mtp reduced cleaved‐caspase 3 protein expression of BM‐EPCs induced by TBHP. The densitometric analysis of all Western blot bands was normalized to the total proteins or GAPDH. *n* = 3 independent experiments. *P < 0.05, **P < 0.01, ***P < 0.005, and ****P < 0.001 versus the indicated group.

The cellular ROS level was significantly increased in a TBHP concentration‐dependent manner, but was significantly attenuated by pre‐treatment with Mtp (Fig. 4A–C). Correspondently, it can clearly see that cell apoptosis, as shown by the results of live/dead, CCK‐8, TUNEL tests and protein analysis of cleaved‐caspase 3 (Fig. 4D–K), was significantly increased in TBHP‐induced high ROS level group, suggesting that ROS accumulation can induce apoptosis. In contrast, cells that were not treated with TBHP exhibited very low levels of apoptosis, indicating Mtp exerts potent protective effects against TBHP‐induced cell apoptosis.

### Mtp inhibits mitochondrial dysfunction‐induced cellular apoptosis

As shown in Fig. S2, TBHP induced mitochondrial dysfunction in a concentration‐dependent manner, as shown by the up‐regulated protein levels of caspase 9, Bax and cytochrome c and decreased Bcl‐2 expression in BM‐EPCs (Fig. S2a–S2c).

**Figure 5:**
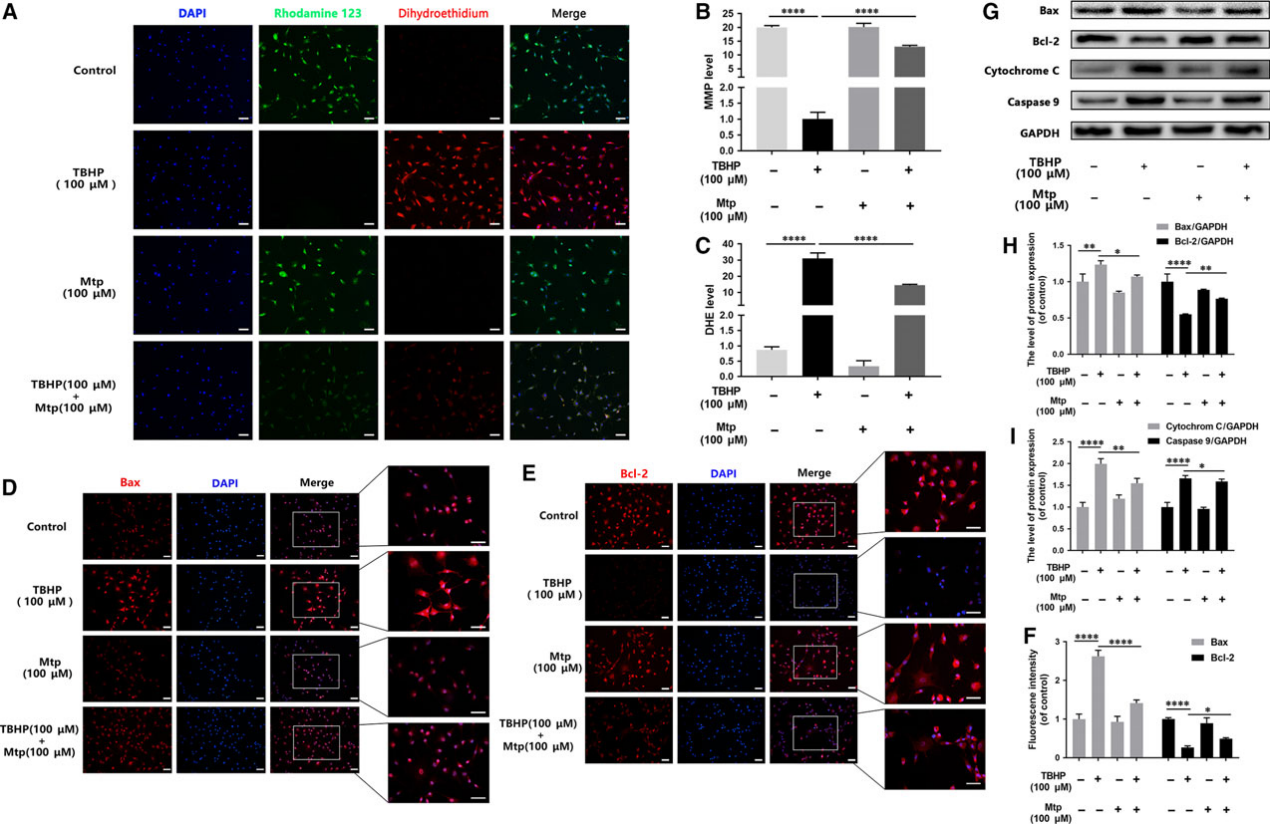
Mtp alleviates oxidative stress‐mediated mitochondrial dysfunction in BM‐EPCs. (**A**–**C**) Fluorescence staining results of mitochondrial membrane potential by rhodamine 123 and ROS production by ROS probe DHE. BM‐EPCs were treated with 100 μM Mtp for 48 hrs and TBHP for 3 hrs and then labelled with fluorescent dyes rhodamine 123, DHE and Hoechst 33258. Representative images were taken from stained BM‐EPCs of indicated groups (scale bar: 30 μm). MMP significantly increased and ROS products decreased in Mtp and/or TBHP‐treated BM‐EPCs; (**D**–**F**) immunofluorescence staining results of Bax and Bcl‐2 in Mtp and/or TBHP‐treated BM‐EPCs (scale bar: 50, 50 μm); (**G**–**I**) Western blot analysis of protein expression of Bax, Bcl‐2, cytochrome c, caspase 9 in BM‐EPCs treated with 100 μM Mtp for 48 hrs and TBHP for 3 hrs. Pre‐treatment with Mtp evidently decreased the release of pro‐apoptotic proteins. The densitometric analysis of all Western blot bands was normalized to the total proteins or GAPDH. *n* = 3 independent experiments. *P < 0.05, **P < 0.01, ***P < 0.005, and ****P < 0.001 versus the indicated group.

Rhodamine 123, a lipophilic cationic probe, was used to assess mitochondrial transmembrane potential (MMP) changes. Figure 5A and B shows that exposure to TBHP rapidly induced MMP dissipation, which can be restored by Mtp pre‐treatment in BM‐EPCs. Furthermore, notably decreased dihydroethidium (DHE) intensity was observed in the Mtp group (Fig. 5C) compared with untreated controls, indicating that mitochondrial dysfunction was related to ROS accumulation, further leading to apoptosis in endothelial cells.

Additionally, significantly increased Bax expression was observed in the cytoplasm of TBHP‐treated cells. Interestingly, BM‐EPCs pre‐treated with Mtp showed substantially attenuated cytoplasmic Bax expression (Fig. 5D). Conversely, Bcl‐2 expression was down‐regulated subjected to TBHP, whereas Mtp pre‐treatment inhibited TBHP‐induced changes in Bcl‐2 expression (Fig. 5E and F). Western blot analysis also supported these findings (Fig. 5G–I). Taken together, these results showed that Mtp suppressed mitochondria‐induced apoptosis by regulating Bcl‐2 family proteins in BM‐EPCs.

### Mtp inhibits oxidative stress‐induced mTOR‐mediated autophagy

**Figure 6:**
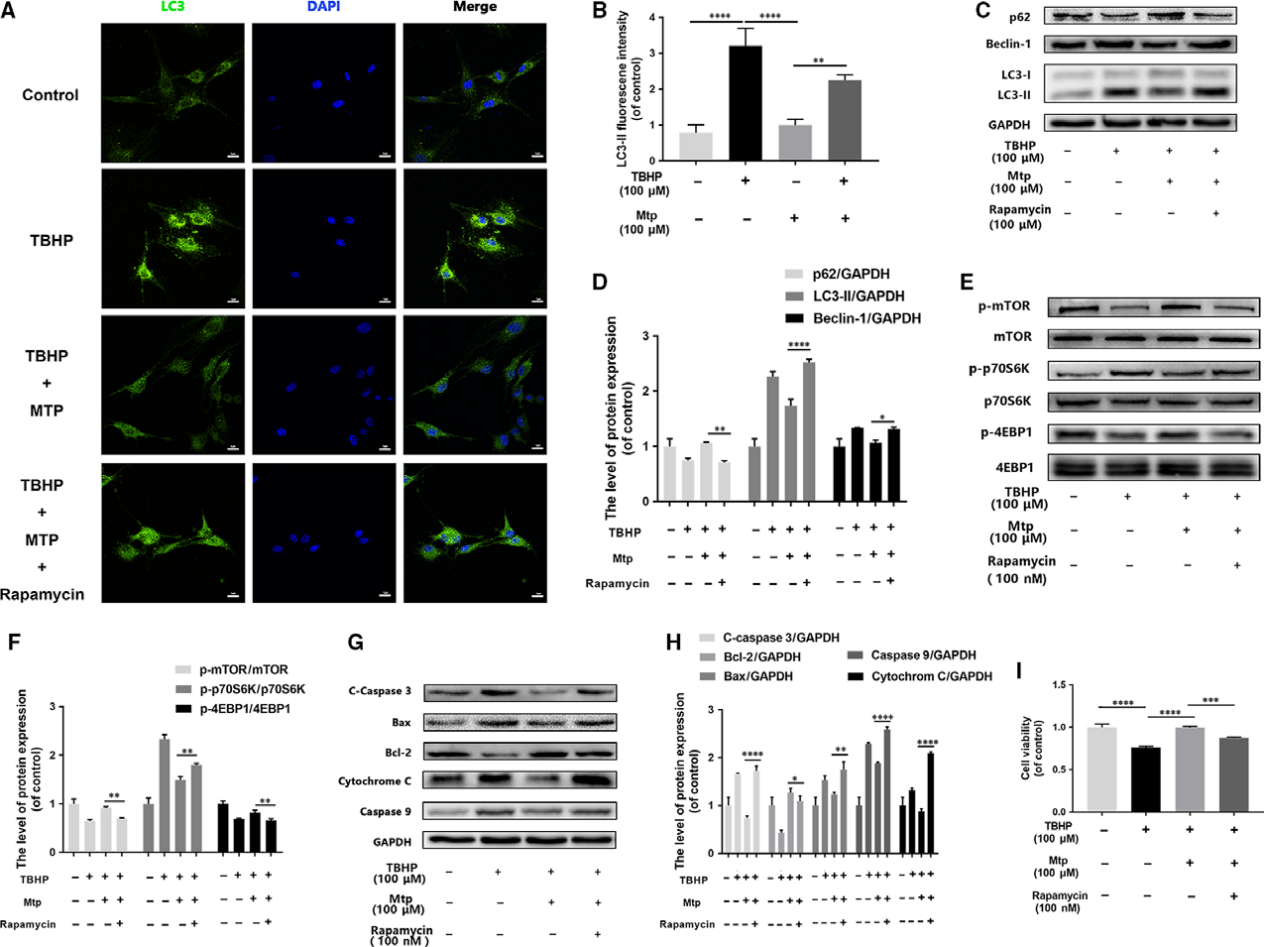
Mtp provides cellular protection against apoptosis in BM‐EPCs *via* mTOR signalling pathways. (**A**,** B**) Immunofluorescence staining images and intensity of LC3‐positive autophagic vesicles (scale bar: 50 μm). LC3 autophagic vesicles were significantly decreased by Mtp pre‐treatment; (**C**–**H**) protein levels of p‐mTOR, p‐p70S6K, p‐4EBP1, SQSTM1/P62, Beclin‐1 and LC3‐II, cleaved‐caspase 3, Bax, Bcl‐2, cytochrome c, caspase 9 in BM‐EPCs treated with 100 nM rapamycin for 2 hrs, 100 μM Mtp for 48 hrs and TBHP for 3 hrs; (**I**) cell viability results by CCK‐8 test of BM‐EPCs treated with 100 nM rapamycin for 2 hrs, 100 μM Mtp for 48 hrs and TBHP for 3 hrs. Cell viability was evidently increased by the Mtp pre‐treatment. The densitometric analysis of all Western blot bands was normalized to the total proteins or GAPDH. *n* = 3 independent experiments. *P < 0.05, **P < 0.01, ***P < 0.005, and ****P < 0.001 versus the indicated group.

Autophagy promotes cell survival in starvation conditions by increasing the available energy; in contrast, excessive autophagy generally causes non‐apoptotic cell death 24, 25. Hence, TBHP‐induced autophagy in BM‐EPCs was investigated. As shown in Fig. S3a, autophagy indicators, such as SQSTM1/P62, Beclin‐1 and the conversion of LC3‐I to LC3‐II, in TBHP‐induced BM‐EPCs were affected in a dose‐dependent manner. Increased TBHP concentrations correlated with higher levels of Beclin‐1 and LC3‐I to LC3‐II conversion as well as lower levels of SQSTM1/P62. As Mtp pre‐treatment exhibited cytoprotective effects against excessive autophagy caused by TBHP‐induced oxidative stress in our study, mTOR signalling, which mediates oxidative stress‐induced autophagy, was evaluated. As shown in Online Fig. S3b, TBHP (100 μM) significantly down‐regulated the levels of p‐mTOR, p‐p70S6K and p‐4EBP1 and affected autophagy marker expression in BM‐EPCs. In contrast, Mtp pre‐treatment restored mTOR protein phosphorylation and the phosphorylation of its two targets, p70S6K and 4EBP1, despite TBHP treatment. Moreover, the THBP‐induced changes in autophagy marker expression were also attenuated by BM‐EPCs pre‐treatment with Mtp (Fig. S3c). Additionally, to further determine whether decreased autophagy enhanced survival or decreased apoptosis rates, this pathway was blocked by the classical autophagy agonist rapamycin. Immunostaining of LC3‐II in TBHP‐treated BM‐EPCs showed increased LC3‐II expression in the cytoplasm, which was reversed by Mtp even with the rapamycin pre‐treatment (Fig. 6A and B). The expression of SQSTM1/p62, LC3‐II and Beclin‐1 was preserved by Mtp pre‐treatment (Fig. 6C and D). As shown in Fig. 6E and F, rapamycin inhibited mTOR/p70S6K/4EBP1 phosphorylation in THBP‐treated cells, which was also partially attenuated by Mtp pre‐treatment. However, rapamycin significantly aggravated oxidative stress‐induced apoptosis and blocked the positive effects of Mtp pre‐treatment as shown in Fig. 6F and H, consistent with the cell viability assay results in Fig. 6I.

To further examine the interplay between autophagy and oxidative stress‐induced cell apoptosis, we pre‐treated BM‐EPCs with 3‐MA, a class III PI3K inhibitor, and CQ, a classical autophagy‐lysosome pathway inhibitor that blocks autophagosome formation. As shown in Fig. S3d, 3‐MA treatment inhibited LC3B‐II and Beclin‐1 expression and accumulated p62/SQSTM1 protein in THBP‐treated cells, whereas CQ inhibited autophagic lysosome formation, augmented LC3‐II expression and restored p62/SQSTM1 and Beclin‐1 expression in THBP‐treated cells. Moreover, significantly down‐regulated Bax and cleaved‐caspase 3 expression and up‐regulated Bcl‐2 expression were observed in the presence of 3‐MA or CQ. Furthermore, the presence of 3‐MA or CQ significantly alleviated oxidative stress‐induced apoptosis as shown by increased cell viability (Fig. S3e). Together, these data indicate that inhibition of excessive autophagy attenuated THBP‐induced cell apoptosis.

### Mtp attenuates cell apoptosis and autophagy through AMPK signalling

**Figure 7:**
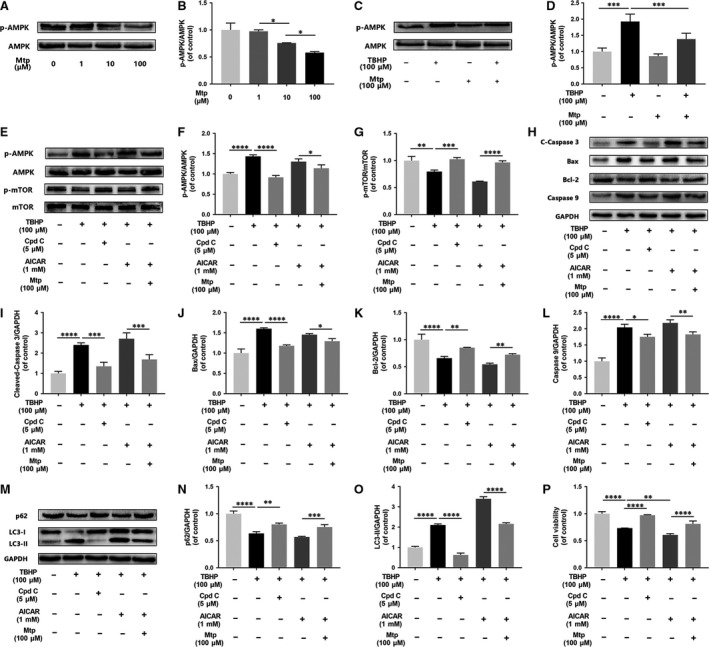
Suppression of AMPK by Mtp attenuates oxidative stress‐mediated cell apoptosis and autophagy. (**A**,** B**) Western blot analysis of expression of p‐AMPK in different doses of Mtp treatment. BM‐EPCs were treated with 1, 10 and 100 μM Mtp. The protein expression of p‐AMPK was decreased after Mtp treatment; (**C**,** D**) Western blot analysis of expression of p‐AMPK after Mtp pre‐treatment. Cells were pre‐treated with 100 μM Mtp followed by TBHP stimulation. Mtp reduced p‐AMPK protein expression of BM‐EPCs induced by TBHP; (**E**–**O**) Western blot analysis of expression of p‐AMPK, p‐mTOR, cleaved‐caspase 3, Bax, Bcl‐2, caspase 9, SQSTM1/P62 and LC3‐II. Cells were pre‐treated with 5 μM compound C or 1 mM AICAR for 2 hrs followed by 100 μM Mtp for 48 hrs and incubated with TBHP for 3 hrs. (**P**) Cell Counting Kit‐8 (CCK‐8) results of BM‐EPCs were treated under the same conditions as above. Cell viability was significantly increased by Cpd C treatment. The densitometric analysis of all Western blot band intensities was normalized to the total proteins or GAPDH. *n* = 3 independent experiments. *P < 0.05, **P < 0.01, ***P < 0.005, and ****P < 0.001 versus the indicated group.

Decreased levels of p‐AMPK were observed when cells were treated with Mtp (Fig. 7A and B), suggesting that Mtp may inhibit the phosphorylation of AMPK to activate mTOR receptors and restore cell viability. The THBP‐induced up‐regulation of p‐AMPK was attenuated by Mtp pre‐treatment (Fig. 7C and D). To further assess the function of AMPK in EPC autophagy and apoptosis following TBHP exposure, we cocultured BM‐EPCs with TBHP, the classical AMPK inhibitor compound C and the AMPK agonist AICAR. As expected, compound C substantially blocked TBHP‐induced AMPK phosphorylation but elevated mTOR phosphorylation (Fig. 7E–G). The increased Bax, cleaved‐caspase 3 and caspase 9 levels induced by TBHP were significantly reversed by compound C. Additionally, significantly higher levels of Bax, cleaved‐caspase 3 and caspase 9 were observed in AICAR‐treated cells compared with the levels in the TBHP group (Fig. 7H–L), suggesting that AMPK phosphorylation is responsible for TBHP‐mediated apoptosis in BM‐EPCs. In addition, cell apoptosis caused by cotreatment with THBP and AICAR was significantly attenuated by Mtp preconditioning, indicating that Mtp may exert cytoprotective effects by regulating the AMPK/mTOR pathway. In contrast to AICAR, compound C down‐regulated LC3‐II accumulation and restored SQSTM1/p62 protein expression after THBP stimulation (Fig. 7M–O), demonstrating that AMPK is required for the activation of oxidative stress‐induced autophagy. Furthermore, compound C and Mtp significantly alleviated TBHP‐induced cell death as shown by cell viability assays (Fig. 7P). Together, these findings suggest that AMPK signalling is upstream of autophagic signalling. Reducing AMPK phosphorylation is a crucial step in the prevention of TBHP‐induced apoptosis and autophagy.

### Mtp accelerates wound healing in rats

**Figure 8:**
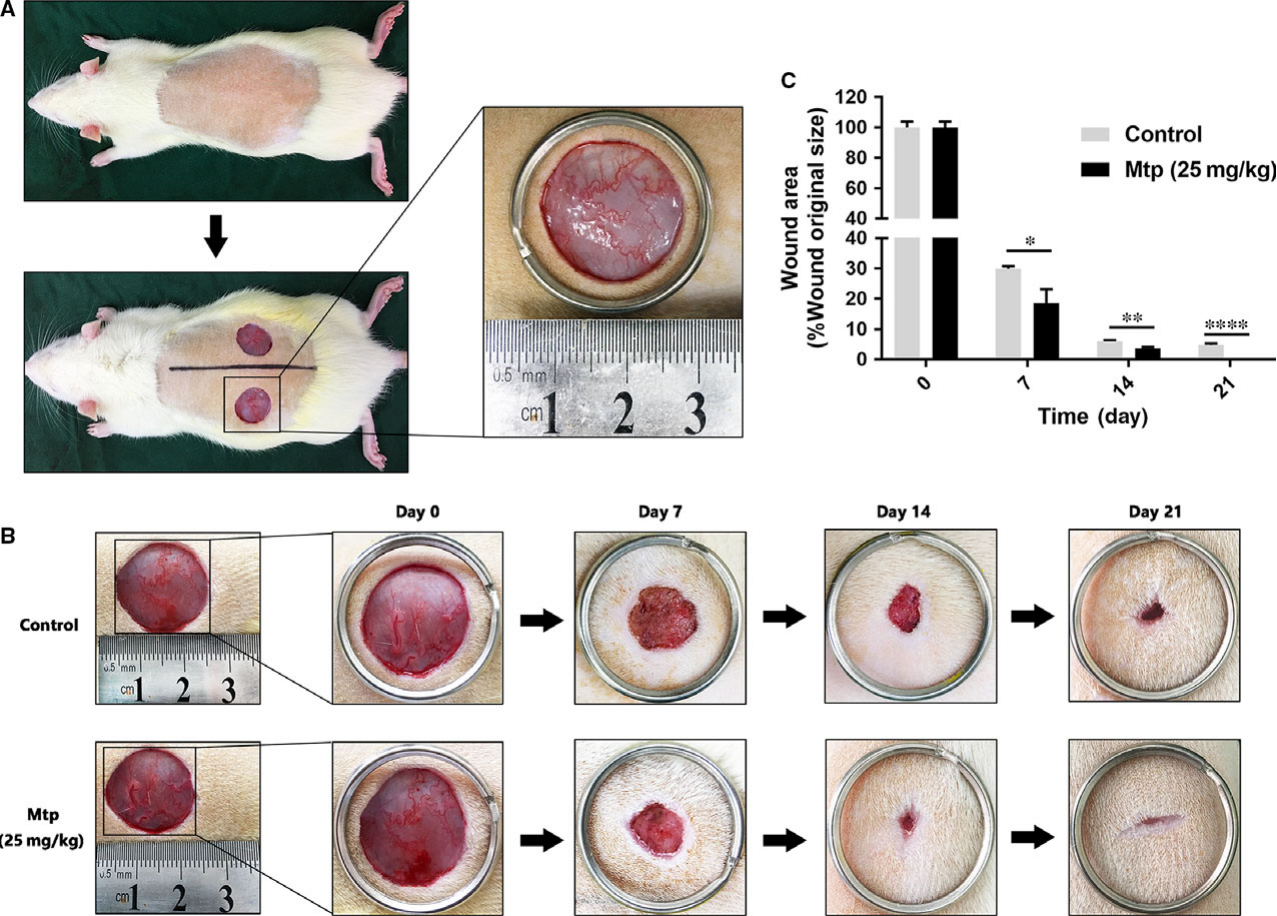
Mtp accelerates wound healing in rats. (**A**) The wound healing model used in this study; (**B**) representative images of healing process in Mtp‐treated rats at different days; (**C**) wound healing rates at different times. Healing rates of full‐thickness cutaneous wounds were significantly increased by the Mtp treatment. *n* = 3 independent experiments. *P < 0.05, **P < 0.01, ***P < 0.005, and ****P < 0.001 versus the indicated group.

Based on the above results, Mtp possesses angiogenic and anti‐apoptotic effects *in vitro*, which may facilitate cutaneous wound healing. Therefore, the wound healing effects of Mtp were investigated in full‐thickness cutaneous wounds in rats. As shown in Fig. 8, wounds treated with Mtp healed more quickly than control (Fig. 8A and B). Wound areas began to shrink and close most rapidly during the first week (Fig. 8C). By day 7, Mtp‐treated wounds achieved nearly 80% closure compared with the 70% wound closure observed in control. On day 14, the healing rate of the Mtp group slowed, but healing still occurred at a significantly higher rate than controls. By day 21, wounds in the Mtp group were completely closed, while some of the control wounds remained unhealed, demonstrating that Mtp accelerated wound healing *in vivo*.

### Mtp enhances collagen deposition and alleviates macrophage infiltration

**Figure 9:**
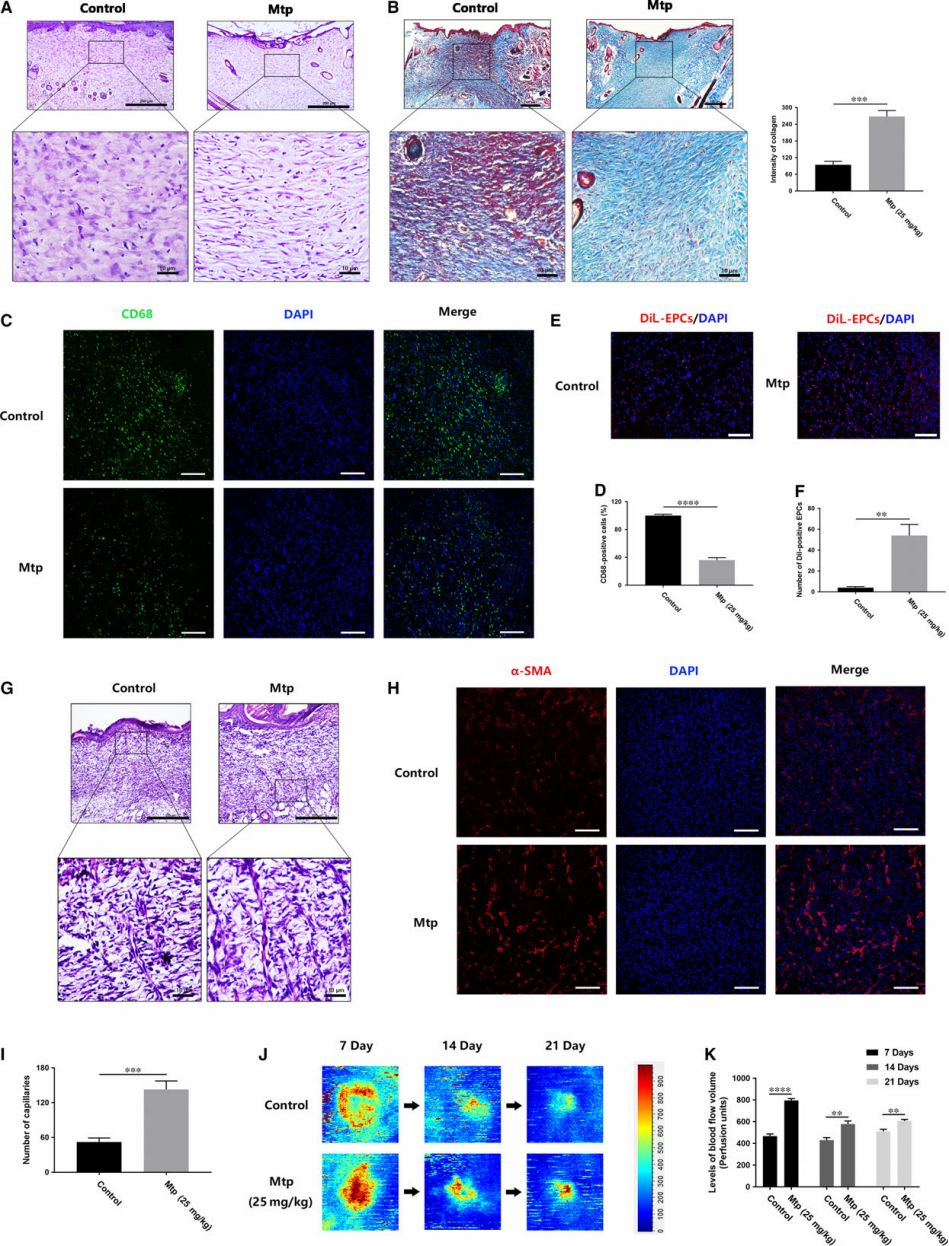
Mtp decreases macrophage infiltration and increases capillary formation. (**A**) H&E staining images of wound tissue treated with Mtp at day 21 (scale bar: 200, 10 μm); (**B**) Masson trichrome staining images of wound tissue treated with Mtp at day 21 and quantification of collagen intensity (scale bar: 200, 10 μm); (**C**,** D**) immunofluorescence staining images and quantification of CD68‐positive macrophages (scale bar: 100 μm); (**E**,** F**) *in vivo* tracing of DiL‐labelled EPCs. Red fluorescence identifies DiL‐labelled EPC and blue fluorescence indicates cell nucleus (scale bar: 100 μm); (**G**) H&E staining images with newly formed blood vessels at day 7 (scale bar: 200, 10 μm); (**H**,** I**) immunofluorescence staining images of α‐SMA (scale bar: 100 μm) and the number of newly formed blood vessels; (**J**) Laser doppler scan photographs of the wounds at days 0, 7, 14 and 21 after injury; (**K**) quantification of the blood flow volume using MoorLDI Review V6.1 software, scale bar: 200 μm. *n* = 3 independent experiments. *P < 0.05, **P < 0.01, ***P < 0.005, and ****P < 0.001 versus the indicated group.

Collagen deposition in the dermis of regenerated skin for different groups at 21 days after injury was evaluated. The arrangement of collagen fibres in the Mtp‐treated wounds appeared more orderly than controls (Fig. 9A and B). Moreover, Mtp‐treated wounds exhibited the significantly higher intensity of collagen deposition than control (Fig. 9B). Consistently, Mtp can promote collagen deposition and remodelling.

Macrophage infiltration in wounds is a sign of inflammation 26, 27, 28. As shown in Fig. 9C and D, the intensity of CD68‐positive stained macrophages was decreased after Mtp treatment, illustrating the decreased macrophage infiltration by Mtp in wounds.

### Mtp promotes EPCs recruitment, blood vessel formation and blood flow to the wound area

To investigate whether EPCs associated with wound healing, we transplanted DiL‐labelled EPCs. As shown in Fig. 9E and F, DiL‐positive EPCs remarkably increased in the wound area, indicating that Mtp could enhance EPC recruitment from circulating blood. To evaluate neovascularization of the wounds, the blood vessels at wound area were characterized by α‐SMA. Significantly higher numbers of newly formed blood vessels were observed in the Mtp group on day 7 compared with the control group (Fig. 9G–I). Moreover, to investigate whether these increased numbers of blood vessels can influence blood flow volume of wounds, blood perfusion was analysed in the wound area. Interestingly, high blood flow was found only around the centre of wound area in the control group, whereas a high signal and increased blood perfusion were observed throughout the wound area in the Mtp group (Fig. 9J and K), suggesting that the increased number of blood vessels led to increased blood perfusion and promoted wound healing.

## Discussion

Wound healing is a biological process involving interactions between cells, skin ECM and growth factors and usually occurs in three stages: inflammation, new tissue formation and remodelling 3. According to recent studies, the external application of growth factors could promote angiogenesis at the site of injury and accelerates wound closure 29. However, high costs, short protein half‐life and undesirable side effects limit their clinical use 30. Thus, development of therapeutic strategies involving effective and pleiotropic proangiogenic drugs is urgently needed. In this study, the TCM Mtp, which has multiple therapeutic effects, was employed, and its angiogenic ability and related mechanisms as well as wound repair capacity were investigated. EPCs can differentiate into endothelial cells, which play important roles in vasculogenesis and angiogenesis 4. Here, a significant proangiogenic effect of Mtp on BM‐EPCs was observed in our study, as shown by enhanced cell proliferation and regulated adhesion, migration and *in vitro* tube formation. Generally, vascular endothelial proliferation is the beginning of angiogenesis, followed by separating from adjacent cells, migration, adherence to the ECM and differentiation 31, 32. According to the present study, Mtp significantly enhanced the proliferation, chemotactic capacity and differentiation of BM‐EPCs in a concentration‐dependent manner. Moreover, the pre‐treatment of Mtp on BM‐EPCs decreased cell–cell adhesion but increased cell–matrix adhesion, which also supported the potent proangiogenic effects of Mtp. On the other hand, growth factors, such as PDGF, bFGF and VEGF, will benefit vascular formation in wound healing. VEGF is a vital modulator for vascular development, which increases the endothelial cell proliferation, survival and migration and promotes angiogenesis 33, 34, and its primary receptor VEGFR‐2 (KDR) mediates the growth and permeability effects of VEGF 35. Our data showed that Mtp enhanced VEGF‐A, VEGFR‐2 and PECAM‐1 expression in a dose‐dependent manner, indicating that the proangiogenic effects of Mtp may derive from the activation of these genes.

According to several studies, protein kinases, including AKT, mTOR, p70S6K and VEGFR‐2 kinase, can regulate angiogenesis 36. Here, treatment with Mtp significantly increased the phosphorylation of mTOR, p70S6K and 4EBP1, and significantly decreased the autophagy level with up‐regulated SQSTM1/p62 and down‐regulated Beclin‐1 levels. Interestingly, Mtp stimulated BM‐EPC chemotaxis and tube formation, which was dramatically attenuated by the autophagy agonist rapamycin, indicating that excessive autophagy suppresses the proangiogenic effects of Mtp on BM‐EPCs. Research showed that oxidative stress induced by exogenous TBHP can trigger autophagy 15. In this study, TBHP was applied to induce oxidative stress and autophagy in BM‐EPCs; as expected, these damages can be attenuated by Mtp pre‐treatment, suggesting the potent protective function of Mtp on oxidative stress.

It has been reported blood vessels were impaired in diabetes mellitus, atherosclerosis and hypertension, which may be related to the presence of oxidative stress and increased concentration of oxidation products 37, 38, 39. Oxidative stress‐mediated apoptosis would lead to ROS formation and the loss of MMP through the mitochondrial pathway 40, 41. Notably, Mtp significantly increased the viability of TBHP‐treated cells by reducing ROS formation. Moreover, decreased MMP, translocated intranuclear proteins (Bax) and released cytochrome c levels in the cytoplasm were found, leading to mitochondrial membrane dysfunction in TBHP‐treated BM‐EPCs 42. Then, the released cytochrome c activates caspase 9, which causes caspase 3 cleavage and eventually apoptosis 43. As shown in the present study, treatment with Mtp prior to TBHP reversed mitochondria‐driven apoptosis by reducing the cytoplasmic pro‐apoptotic Bax protein expression and increasing anti‐apoptotic Bcl‐2 protein levels, restoring MMP but inhibiting cytochrome c release in BM‐EPCs, indicating that Mtp exerted its protective function of antioxidative stress‐induced apoptosis through mitochondrial pathway.

**Figure 10:**
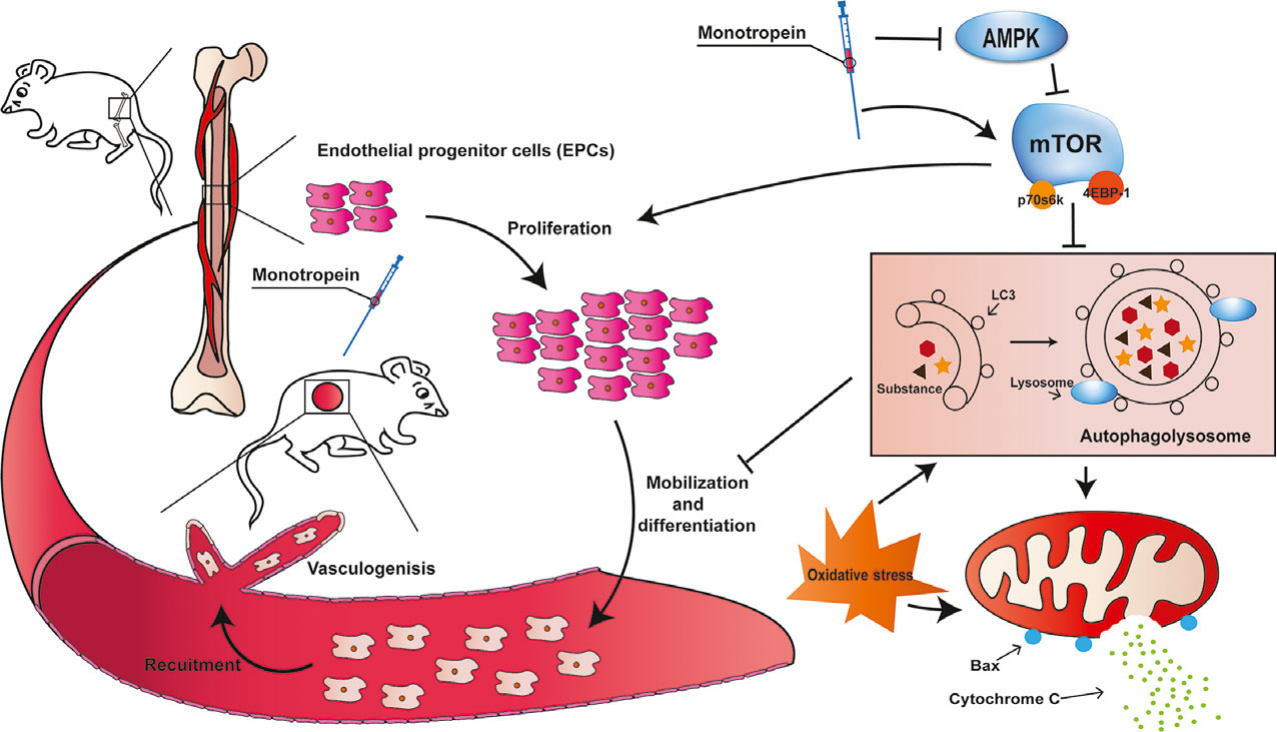
Schematic of Mtp enhances wound healing. Mtp promotes proliferation, mobilization, differentiation and recruitment in BM‐EPCs, together leading to promote angiogenesis. Mtp also mitigates oxidative stress‐induced apoptosis *via* AMPK/mTOR pathway through suppression autophagy and mitochondrial apoptosis of EPCs, together leading to faster vascularization and wound healing in rats.

mTOR is a crucial cell signalling receptor involved in multiple cellular activities, including proliferation, migration and cell survival 44, 45. Rapamycin, an inhibitor of mTOR, significantly abolished the protective effects of Mtp on BM‐EPCs, indicating a vital role for mTOR phosphorylation in this process. Moreover, mitochondrial metabolism is associated with the mTOR complex, regulating both resting oxygen consumption and the oxidative capacity. Oxidative stress‐related products, such as ROS and superoxides, enhanced autophagy and induced cell death 46, 47. Moreover, autophagy induced by high glucose 48 or the Alzheimer disease‐associated peptide 49, can impair angiogenesis in EPCs. Consistent with prior studies, our results indicated that TBHP stimulated autophagosome formation in a dose‐dependent manner in BM‐EPCs, which, interestingly, was attenuated by Mtp, as shown by reduced numbers of autophagic vacuoles and levels of autophagy‐related proteins. However, mTOR dephosphorylation aggravated the endogenous endothelial‐protective pathways against oxidative stress in BM‐EPCs, as indicated by the increased cell death observed following rapamycin treatment but decreased cell death following co‐administration of the autophagy inhibitors 3‐MA and CQ. In summary, TBHP‐induced autophagy could cause EPC dysfunction, oxidative stress‐mediated mitochondrial damage and angiogenic dysfunction (Fig. 10).

Emerging reports have suggested that AMPK is vital for cell survival and can be activated by ROS, leading to mTOR phosphorylation and autophagy induction 50. Consistent with our results, oxidative stress‐induced ROS‐dependent AMPK stimulation is critical for autophagy activation. According to a previous study, a MEK/ERK module regulated autophagy through the AMPK‐MEK/ERK‐TSC‐mTOR signalling pathway 51. Here, TBHP induced autophagy *via* AMPK phosphorylation and mTOR signalling. Based on these results, AMPK, an upstream factor of mTOR, is an essential target of oxidative stress and may play a role in mediating the protective effects of Mtp in BM‐EPCs.

Meanwhile, we present *in vivo* evidence indicating Mtp promotes cutaneous wound healing in SD rats. Increased levels of fibroblasts, blood vessels and collagen deposition were observed in Mtp‐treated wounds, which would facilitate wound healing *in vivo*. Meanwhile, H&E staining results showed that blood vessels containing red staining appeared in the Mtp‐treated wounds (Fig. 8E), which is a sign of mature blood vessels with red blood cells in the lumen. Then the immunostaining of α‐SMA, a marker of smooth muscle cells that dominate the middle layer of mature blood vessel, was used to reveal the maturity of vessels because α‐SMA can be a marker of mature vessels according to several studies 52, 53, 54. Results showed more numbers of α‐SMA‐positive stained cells in Mtp‐treated wounds, suggesting that Mtp can not only promote the blood vessels formation, but also accelerate their maturity to facilitate wound healing. Laser Doppler analysis results also confirmed the higher blood flow of functional blood vessels, which indirectly suggested the faster formation and maturity of blood vessels. These results also confirmed the *in vitro* proangiogenic effect of Mtp, indicating that fast angiogenesis can be achieved *in vivo* and further accelerate wound healing.

Additionally, reduced macrophage infiltration was also found in the Mtp group at day 7. It is well known that certain amount of inflammatory cells at the early stage could benefit would healing by secreting growth factors and stimulate the proliferation of other related cells 55, 56; however, chronic inflammation causes substantial tissue damage and promotes a continuous recruitment of inflammatory cells, thereby exacerbating damage and healing 57. Here, the macrophages were activated with a mild status, which can help the secretion of growth factors and help wound healing without evoking chronic inflammation. Studies also showed the interactions between EPCs in wound areas and polymorphonuclear leucocytes (PMNLs), which derived ROS could induce EPC damage and further impair wound healing 58, suggesting that the reduced inflammatory response of wound area may be due to the function of EPCs activated by Mtp. Interestingly, studies have been reported that EPCs can be mobilized endogenously by tissue ischaemia or exogenously by cytokine treatment 59, 60. Our study showed that Mtp enhanced the recruitment of EPCs to the wound area. Further research is needed to be performed to investigate the specific function and mechanism of BM‐EPCs *in vivo*.

As shown in the present study, TBHP‐induced autophagy leads to BM‐EPC damage, which contradicts the findings of another study that reported the ability of autophagy to protect EPCs from oxidative stress 61. This discrepancy may derive from differences in treatment, as TBHP may cause more severe oxidative stress than other treatments, leading to the excessive activation of autophagy and non‐apoptotic programmed cell death (also called autophagic cell death), whereas mild treatments such as ox‐LDL may stimulate moderate autophagy that helps EPCs survive under mild conditions. However, it remains unclear how Mtp inhibits autophagy through the AMPK pathway; additionally, the molecular mechanisms underlying angiogenesis promotion and apoptosis reduction *via* autophagy suppression remain unknown. Further studies are required to investigate these issues and evaluate the possible effects of Mtp on wound healing in human patients.

In summary, our study provides evidence for Mtp‐mediated autophagy inhibition through AMPK/mTOR signalling in BM‐EPCs, leading to increased angiogenic differentiation and a potent anti‐apoptotic effect to combat oxidative stress; these effects were abrogated by the autophagy activator rapamycin. These findings suggest the promising therapeutic potential of Mtp in endothelial injury‐mediated vascular diseases, particularly for patients with skin wounds.

## Conflicts of interest

The authors declare no conflict of interest.

## Supporting information


**Figure S1.** Morphology and characterization of EPCs from rat bone marrow cells.
**Figure S2.** TBHP induces mitochondria dysfunction‐mediated apoptosis in BM‐EPCs.
**Figure S3.** Mtp prevents BM‐EPCs apoptosis via blocks autophagosomeformation.
