#  

**DOI:** 10.1111/jcmm.16995

**Published:** 2021-11-07

In Chenggui Wang,^1^ the published version contains errors in Figures 1D, E, 3B, F, H, 6E and 7E. The correct figures are shown below. All results and conclusions remain intact. The authors apologize for the errors.

1

**FIGURE 1:**
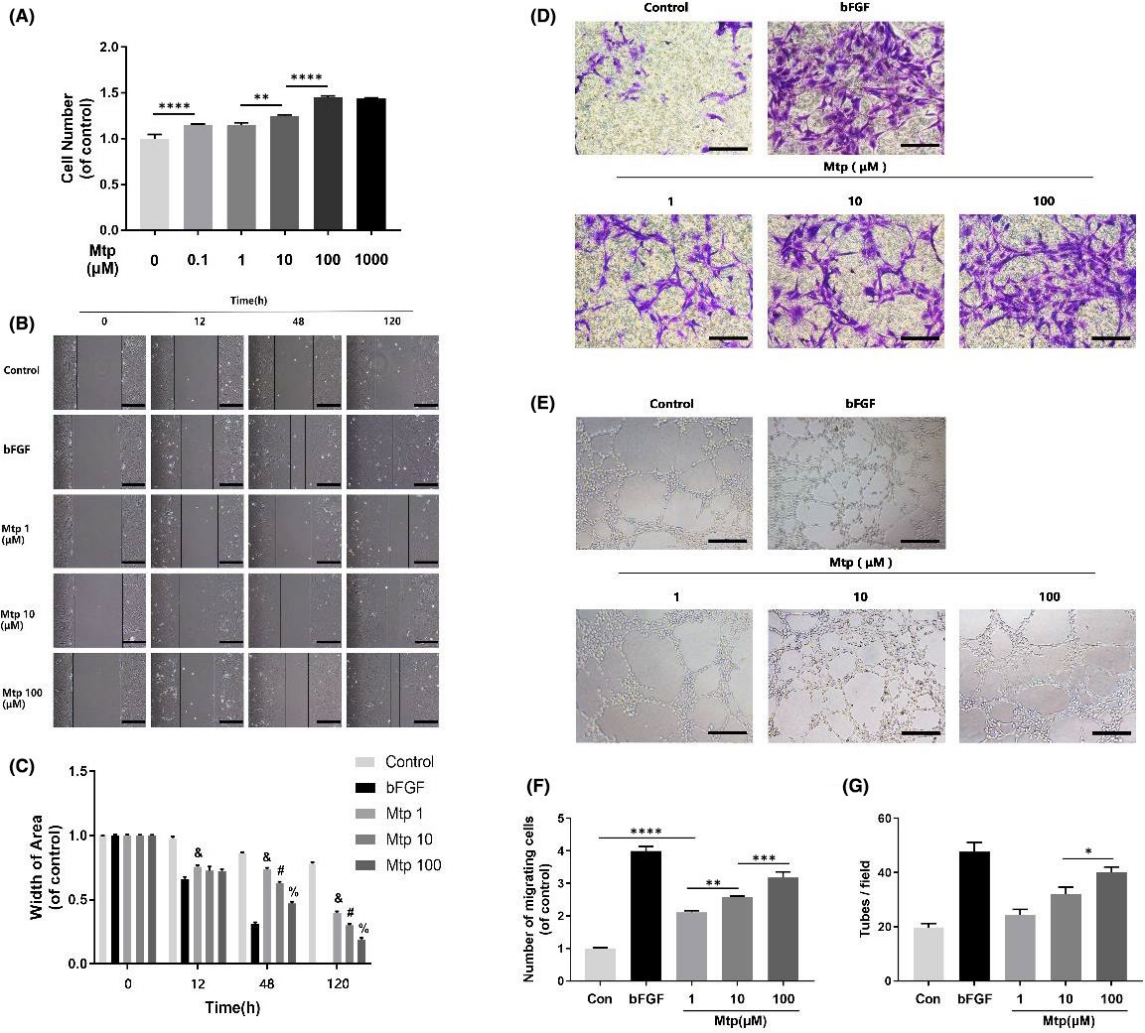
Effect of Mtp on cellular proliferation, migration, recruitment and tube formation of BM‐EPCs. (A) Cell proliferation results of BM‐EPCs treated with different concentrations of Mtp for 48 h. Cells proliferated evidently faster after Mtp treatment; (B, C) Cell migration regulated by Mtp treatments. Scratch assay showed that EPCs migrated evidently faster in Mtp‐treated group (scale bar: 200 μm); Data are presented as mean ± SD, & *p* < 0.05 versus the control group, # *p* < 0.05 versus the 1 μM Mtp‐treated group, % *p* < 0.05 versus the 10 μM Mtp‐treated group; (D, F) Transwell chemotaxis assay results of BM‐EPCs with different treatments. BM‐EPCs were treated with PBS (control), 50 ng/ml bFGF and 1, 10 and 100 µM Mtp in the lower chamber for 3 h incubation. Numbers of migrated cells were quantified by counting cells in 10 random fields using an inverted microscope (scale bar: 50 μm). The migration of BM‐EPCs was enhanced after Mtp treatment; (E, G) In‐vitro tube formation results of BM‐EPCs treated by Mtp. Cells were grown on MatrigelTM for 6 h under normal growth conditions, five independent fields were assessed for each well and the number of tubes were determined (scale bar: 100 μm). The tube formation ability of BM‐EPCs was improved after Mtp treatment. *n* = 3 independent experiments. *p < 0.05, **p < 0.01, ***p < 0.005 and ****p < 0.001 versus the indicated group

**FIGURE 3:**
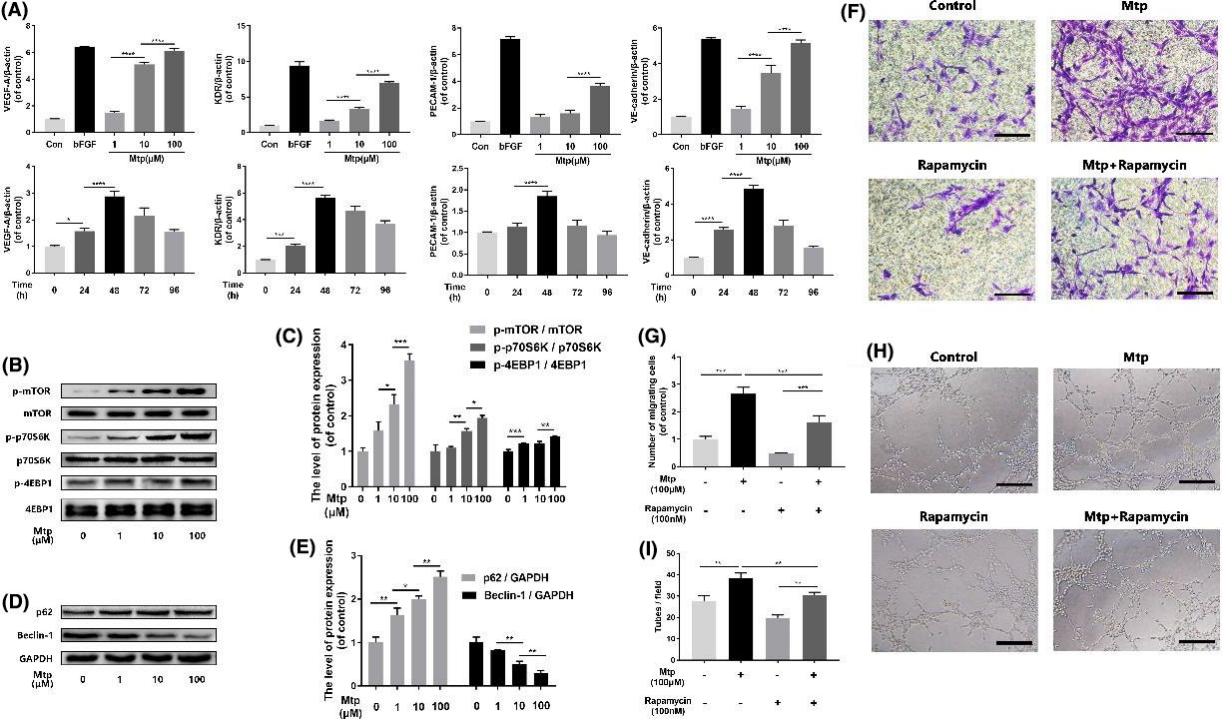
mTOR pathway regulates chemotaxis and capillary‐tube formation capacity of BM‐EPCs. (A) Gene expression of VEGF, KDR, PECAM‐1 and VE‐cadherin in Mtp‐treated BM‐EPCs. Cells were cultivated with 1, 10 and 100 µM Mtp or bFGF (50 ng/ml) for 48 h and treated with 100 µM Mtp for 24, 48, 72 and 96 h. Gene levels were assessed via qRT‐PCR and normalized to β‐actin, related gene were upregulated by Mtp at 48 h or less; (B, C, D, E) Western blot analysis of p‐mTOR, p‐p70S6K, p‐4EBP1, SQSTM1/P62 and Beclin‐1 in different dose of Mtp‐treated BM‐EPCs for 48 h. Mtp evidently increased mTOR pathway proteins and decreased autophagy level; (F, G) Cell chemotaxis regulated by the treatments of rapamycin and/or Mtp. BM‐EPCs were treated with 100 nM rapamycin for 2 h prior to treatment with Mtp for 48 h. The numbers of migrated cells were quantified by performing cell counts of 10 random fields (scale bar: 50 μm); (H, I) In‐vitro tube formation results of BM‐EPCs treated by rapamycin and/or Mtp (scale bar: 100 μm); The densitometric analysis of all Western blot bands was normalized to the total proteins or GAPDH. n = 3 independent experiments. *p < 0.05, **p < 0.01, ***p < 0.005 and ****p < 0.001 versus the indicated group

**FIGURE 6:**
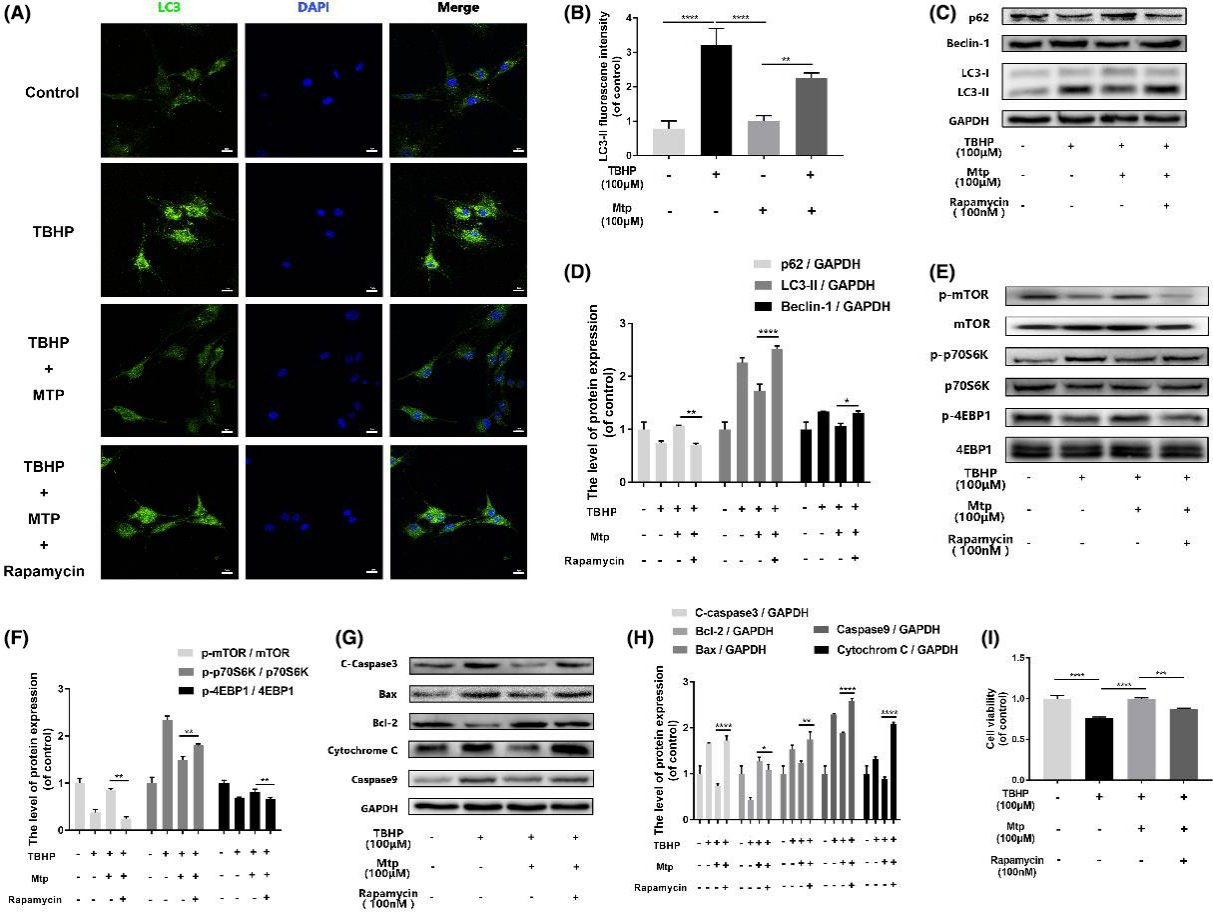
Mtp provides cellular protection against apoptosis in BM‐EPCs via mTOR signalling pathways. (A, B) Immunofluorescence staining images and intensity of LC3 positive autophagic vesicles (scale bar: 50 μm). LC3 autophagic vesicles were significantly decreased by Mtp pretreatment; (C–H) Protein levels of p‐mTOR, p‐p70S6K, p‐4EBP1, SQSTM1/P62, Beclin‐1 and LC3‐II, Cleaved‐caspase3, Bax, Bcl‐2, Cytochrome C, Caspase9 in BM‐EPCs treated with 100 nM rapamycin for 2 h, 100 µM Mtp for 48 h and TBHP for 3 h; (I) Cell viability results by CCK‐8 test of BM‐EPCs treated with 100 nM rapamycin for 2 h, 100 µM Mtp for 48 h and TBHP for 3 h. Cell viability was evidently increased by the Mtp pretreatment. The densitometric analysis of all Western blot bands was normalized to the total proteins or GAPDH. *n* = 3 independent experiments. *p < 0.05, **p < 0.01, ***p < 0.005 and ****p < 0.001 versus the indicated group

**FIGURE 7:**
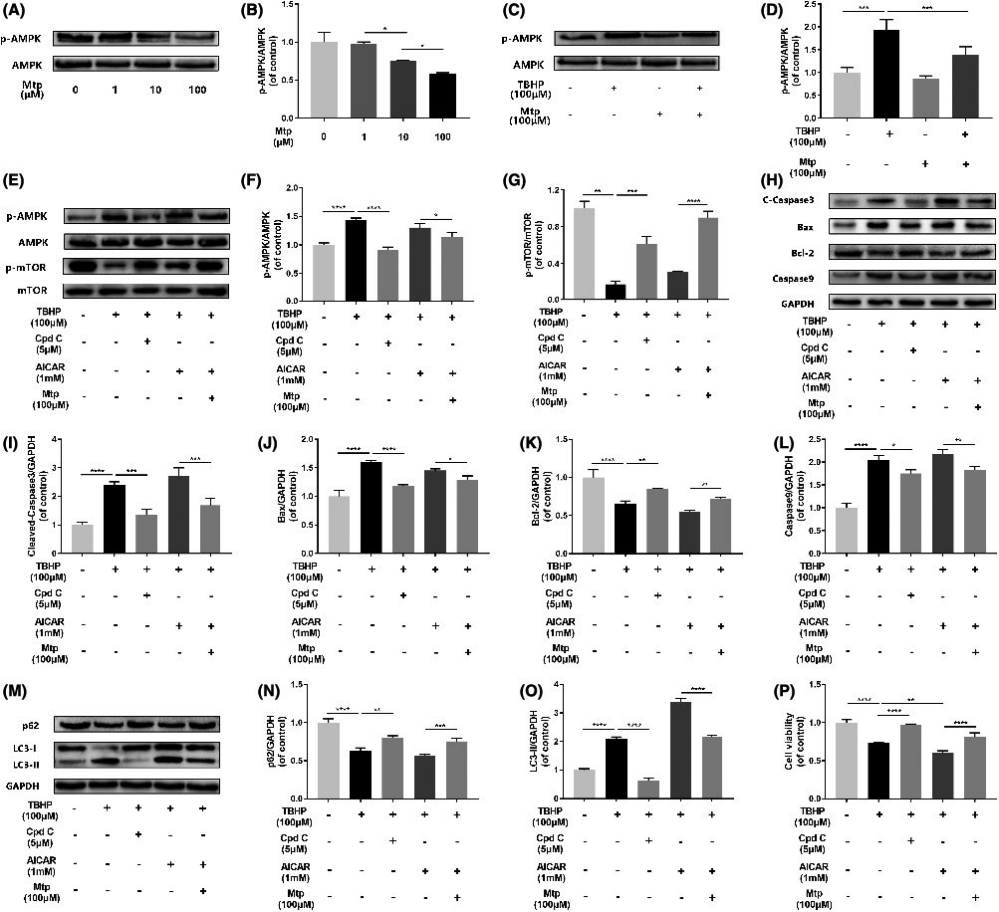
Suppressed of AMPK by Mtp attenuates oxidative stress‐mediated cell apoptosis and autophagy. (A, B) Western blot analysis of expression of p‐AMPK in different dose of Mtp treatment. BM‐EPCs were treated with 1, 10, 100 μM Mtp. The protein expression of p‐AMPK was decreased after Mtp treatment; (C, D) Western blot analysis of expression of p‐AMPK after Mtp pretreatment. Cells pretreated with 100 μM Mtp followed by TBHP stimulation. Mtp‐reduced p‐AMPK protein expression of BM‐EPCs induced by TBHP; (E–O) Western blot analysis of expression of p‐AMPK, p‐mTOR, cleaved‐caspase3, Bax, Bcl‐2, caspase9, SQSTM1/P62 and LC3‐II. Cells were pretreated with 5 µM compound C or 1 mM AICAR for 2 h followed by 100 µM Mtp for 48 h and incubated with TBHP for 3 h. (P) Cell Counting Kit‐8 (CCK‐8) results of BM‐EPCs were treated under the same conditions as above. Cell viability was significantly increased by Cpd C treatment. The densitometric analysis of all Western blot band intensities was normalized to the total proteins or GAPDH. *n* = 3 independent experiments. *p < 0.05, **p < 0.01, ***p < 0.005 and ****p < 0.001 versus the indicated group

## References

[1] Wang C , Mao C , Lou Y , et al. Monotropein promotes angiogenesis and inhibits oxidative stress‐induced autophagy in endothelial progenitor cells to accelerate wound healing. J Cell Mol Med. 2018;22:1583‐1600. doi:10.1111/jcmm.13434. 29278309 PMC5824424

