# Supplementary material for: Monotropein promotes angiogenesis and inhibits oxidative stress‐induced autophagy in endothelial progenitor cells to accelerate wound healing
Source: J Cell Mol Med. 2017 Dec 26;22(3):1583–600. doi: 10.1111/jcmm.13434 (PMC5824424; doi:10.1111/jcmm.13434)
Supplement: Supplementary file 1 — Figure S1. Morphology and characterization of EPCs from rat bone marrow cells. Figure S2. TBHP induces mitochondria dysfunction‐mediated apoptosis in BM‐EPCs. Figure S3. Mtp prevents BM‐EPCs apoptosis via blocks autophagosomeformation. [file JCMM-22-1583-s001.pdf]

Fig. S1

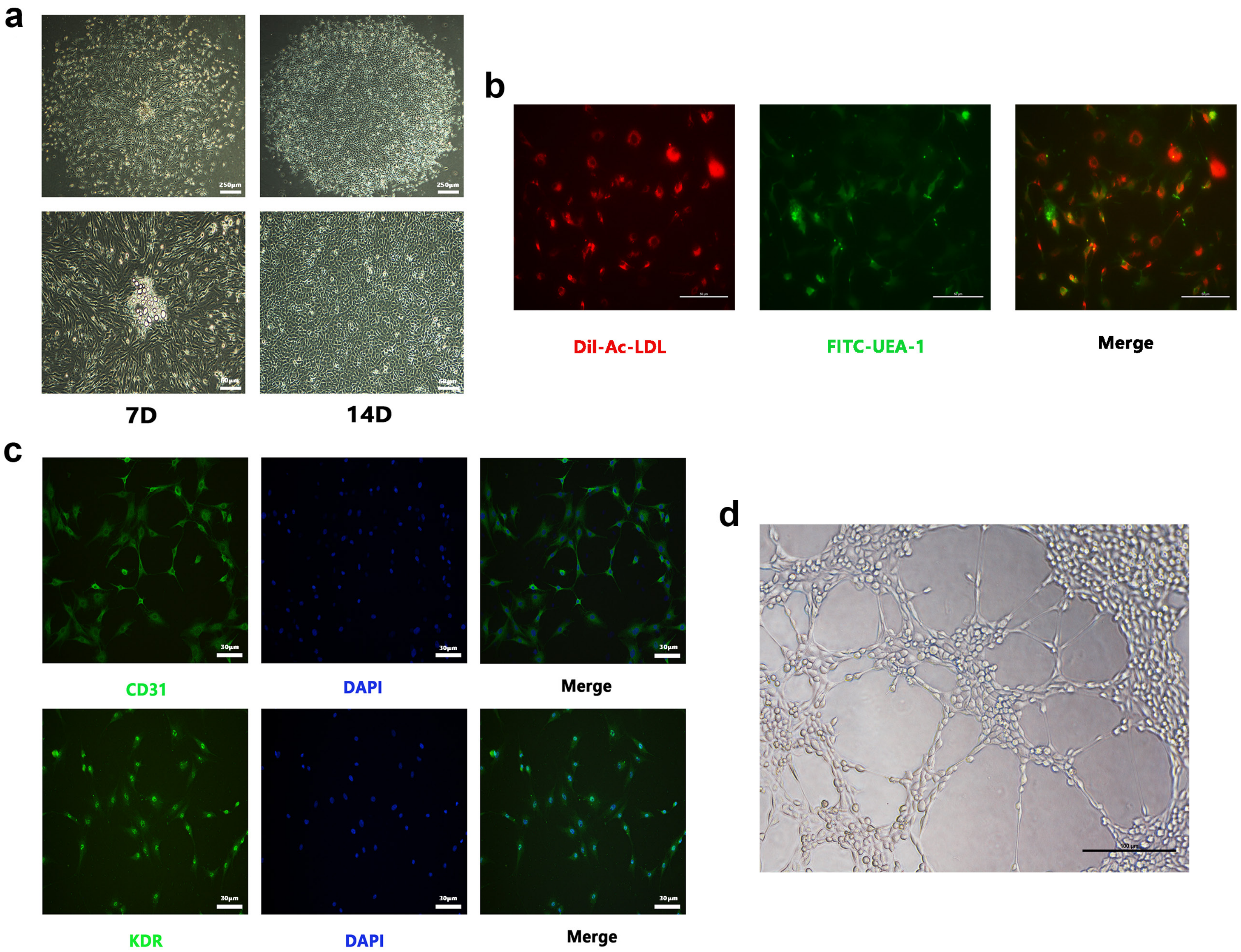

Figure S1. Morphology and characterization of EPCs from rat bone marrow cells. (a) The morphology of EPCs in different growth periods. 7 days-cultured EPCs formed apparent colonies, 14 days cultured EPCs grown to confluence showed a cobblestone-like monolayer (scale bar: 250 μm, 60 μm). **(b)** Immunofluorescence double-staining results of Dil-Ac-LDL and lectin. Most cells were shown to endocytose Dil-Ac-LDL (red), and simultaneously bind FITC-conjugated UEA-1 lectin (green) and double positive cells were identified as differentiating EPCs (scale bar: 50 μm); **(c)** Immunofluorescence staining results of CD31 and KDR. The representative CD31-positive and KDR-positive cells were identified as EPCs (scale bar: 30 μm); **(d)** *In vitro* tube formation results of BM-EPCs. Cells were grown on Matrigel^{TM} for 6 h under normal growth conditions and capillary tube formation was observed under an inverted light microscope (scale bar: 100 μm).

Fig. S2

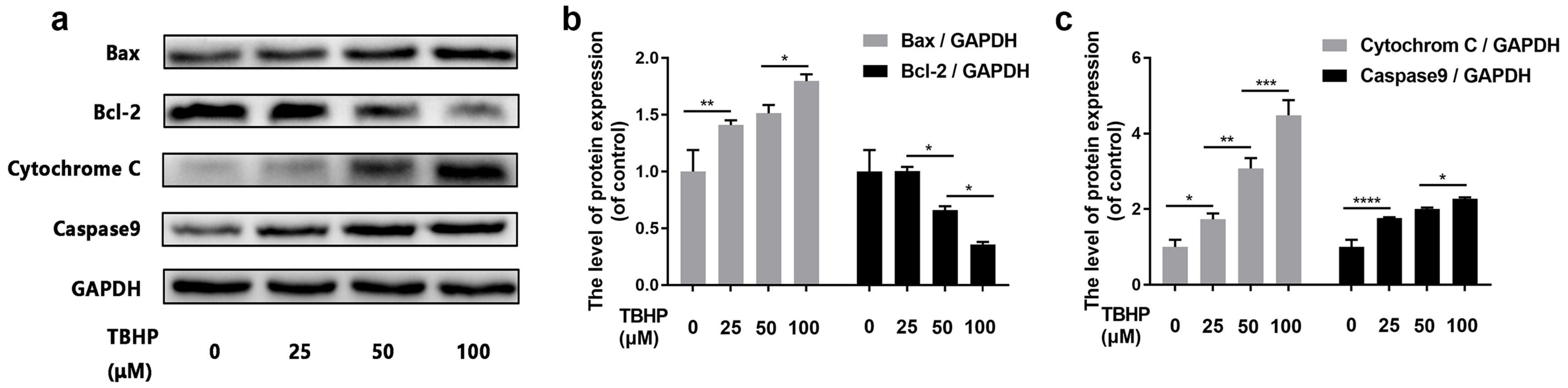

Figure S2. TBHP induces mitochondria dysfunction-mediated apoptosis in BM-EPCs. (a, b, c) Western blot analysis of apoptotic related protein expression of Bax, Bcl-2, Cytochrome C, Caspase 9 in BM-EPCs treated by TBHP, and TBHP evidently increased the release of pro-apoptotic proteins. The densitometric analysis of all Western blot band intensities was normalized to the total proteins or GAPDH. Data are presented as mean ± SD, n=3 independent experiments. Significant differences between the treatment and control groups are indicated as *P < 0.05, **P < 0.01, ***P < 0.005, ****P < 0.001.

Fig. S3

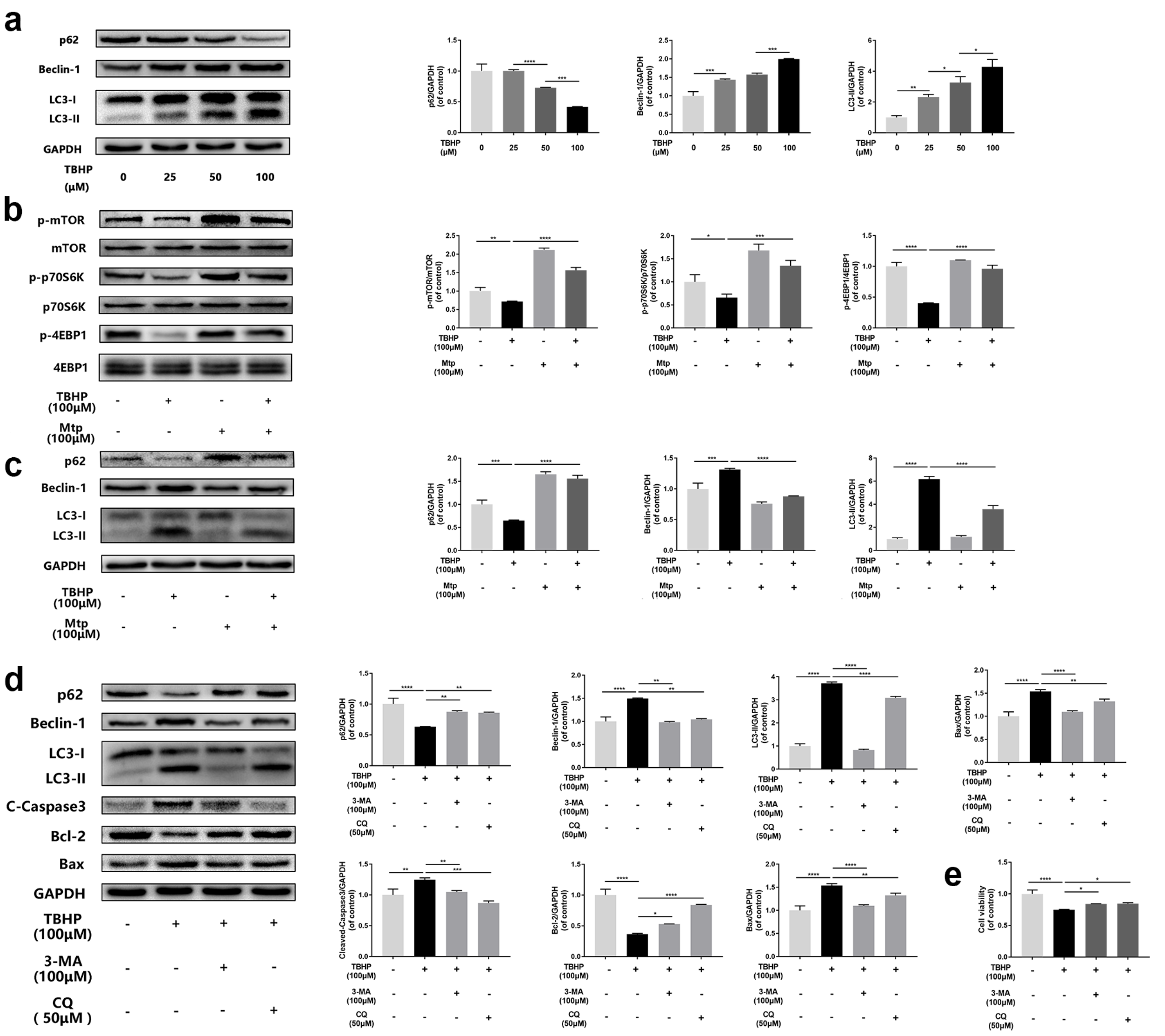

Figure S3. Mtp prevents BM-EPCs apoptosis via blocks autophagosome formation. (a) Western blot analysis of protein expression of SQSTM1/P62, Beclin-1 and LC3-II in BM-EPCs treated with different dose of TBHP. TBHP markedly increased the expression of autophagy-related proteins; **(b, c)** Protein levels of p-mTOR, p-p70S6K, p-4EBP1, SQSTM1/P62, Beclin-1 and LC3-II in BM-EPCs treated with 100 μM of Mtp for 48 h and TBHP for 3 h; **(d)** Western blot analysis of protein expression of SQSTM1/P62, Beclin-1, LC3-II, cleaved-caspase 3, Bcl-2 and Bax in BM-EPCs treated with different autophagy inhibitors. Cells were pretreated with 100 μM 3-MA or 50 μM CQ for 2 h, and then incubated with TBHP for 3 h; **(e)** Cell viability results of BM-EPCs treated with different autophagy inhibitors. Cell viability tested by CCK-8 assay was evidently increased by both 3-MA and CQ pretreatment. The densitometric analysis of all Western blot band intensities was normalized to the total proteins or GAPDH. Data are presented as mean ± SD, n=3 independent experiments. Significant differences between the treatment and control groups are indicated as *P < 0.05, **P < 0.01, ***P < 0.005, ****P < 0.001.
